# Synergistic effects of lipopolysaccharide and rotenone on dopamine neuronal damage in rats

**DOI:** 10.1111/cns.14180

**Published:** 2023-03-21

**Authors:** Jing‐Yi He, Dai‐Di Li, Qian Wen, Ting‐Yang Qin, Hong Long, Shi‐Bin Zhang, Feng Zhang

**Affiliations:** ^1^ Key Laboratory of Basic Pharmacology of Ministry of Education and Joint International Research Laboratory of Ethnomedicine of Ministry of Education and Key Laboratory of Basic Pharmacology of Guizhou Province and Laboratory Animal Center Zunyi Medical University Zunyi Guizhou China

**Keywords:** lipopolysaccharide, neuroinflammation, oxidative stress, Parkinson's disease, rotenone

## Abstract

**Introduction:**

The etiology of Parkinson's disease (PD) is still unknown. Until now, oxidative stress and neuroinflammation play a crucial role in the pathogenesis of PD. However, the specific synergistic role of oxidative stress and neuroinflammation in the occurrence and development of PD remains unclear.

**Methods:**

The changes in motor behavior, dopamine (DA) neurons quantification and their mitochondrial respiratory chain, glial cells activation and secreted cytokines, Nrf2 signaling pathway, and redox balance in the brain of rats were evaluated.

**Results:**

Lipopolysaccharide (LPS)‐induced neuroinflammation and rotenone (ROT)‐induced oxidative stress synergistically aggravated motor dysfunction, DA neuron damage, activation of glial cells, and release of related mediators, activation of Nrf2 signaling and destruction of oxidative balance. In addition, further studies indicated that after ROT‐induced oxidative stress caused direct damage to DA neurons, LPS‐induced inflammatory effects had stronger promoting neurotoxic effects on the above aspects.

**Conclusions:**

Neuroinflammation and oxidative stress synergistically aggravated DA neuronal loss. Furtherly, oxidative stress followed by neuroinflammation caused more DA neuronal loss than neuroinflammation followed by oxidative stress.

## Graphical abstract

Neuroinflammation combined with oxidative stress aggravated dopamine neuronal loss.

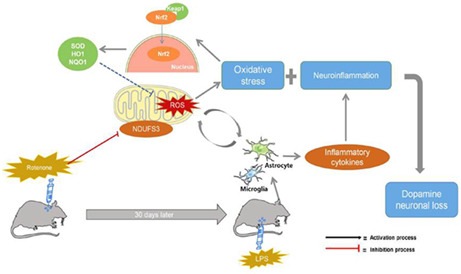

## 1 INTRODUCTION

Parkinson's disease (PD) is one of the most common degenerative diseases in the central nervous system (CNS), with high prevalence rate and even a tendency to low age.^1^ PD is major characterized by progressive loss of dopaminergic (DA) neurons in the substantia nigra (SN) of midbrain. With the development of PD, it is manifested as rest tremor, rigidity, and dyskinesia, which severely affects the normal life of patients.^2^ However, the pathogenesis of PD is still unclear. Until now, oxidative stress and neuroinflammation are considered to be important inducing factors.

Oxidative stress, known as a potential cause of cell dysfunction and eventual death, plays a non‐negligible role in complex neurodegenerative cascades. Normally, cells in the body are dependent on a series of balanced redox reactions that produce energy and take nutrients to synthesize the necessary cellular components to support the occurrence of various biological functions.^3^ When the redox balance in the body is destroyed, an imbalance in the biochemical processes that produce and remove reactive oxygen species (ROS), known as antioxidant cascade reactions, occurs. When excess ROS accumulates, the oxidative stress response in cells is activated.^4^ As an important organelle to maintain the normal function of cells, the mitochondria are injured by oxygen toxicity by a large amount of ROS in the state of oxidative stress, resulting in respiratory chain dysfunction.^5^ Mitochondrial respiratory chains are located on the mitochondrial inner membrane and consist of five complexes, in which electron leakage at mitochondrial complex I releases individual electrons to oxygen and further produces superoxide radicals, a process exacerbated by damage to mitochondrial function.^6^, ^7^ Inhibition of mitochondrial complex I activity in DA neurons leads to the increased ROS production and subsequent oxidative damage to proteins, lipids, and DNA.^8^ NADH: Ubiquitin oxidoreductase core subunit S3 (NDUFS3), a subunit of mitochondrial complex I, is introduced into the mitochondria after nuclear transcription, and this process is down‐regulated in dysfunctional mitochondria.^9^ Rotenone (ROT) is an inhibitor of mitochondrial NADH dehydrogenase, a commonly used natural insecticide that is highly lipophilic and easily penetrates the blood–brain barrier (BBB) into the brain. It has been confirmed that rats exposed to ROT for a long time showed the main characteristics of PD, including loss of DA neurons and motor dysfunction, which is now widely used to induce mammalian PD models.^10^, ^11^ Due to the inherent threat of oxidative stress, mitochondria are often associated with antioxidant defense mechanisms to protect their redox environment. This task is performed by superoxide dismutase (SOD), an antioxidant function that prevents neurodegenerative changes in animal models of PD.^12^, ^13^ Nuclear factor E2‐related factor 2 (Nrf2) is a transcription factor that is sensitive to redox states and maintains cellular redox homeostasis by regulating transcription of antioxidant response elements and expression of antioxidant defense enzymes.^14^, ^15^ Under normal circumstances, Nrf2 binds to Kelch‐like ECH‐Associated Protein 1(Keap1) in the cytoplasm and is not actively transported into the nucleus. However, Nrf2 is stimulated with elevated ROS levels and the stability of binding to Keap1 is disrupted, causing its release and transfer to the nucleus of the cell.^16^ Antioxidant response element (ARE) is a cis‐acting element.^17^ After entering the nucleus, Nrf2 binds to ARE and transcribes downstream genes through relevant activation procedures, thereby activating the expression of heme oxygenase‐1 (HO‐1) and NAD(P)H quinone dehydrogenase 1 (NQO1), generating antioxidant activity.^18^ Therefore, Nrf2 pathway is considered to be an effective way to regulate ROS. Currently, neuroprotective pathways regulated by Nrf2 in astrocytes of the brain have been reported to prevent neuronal oxidative stress.^19^ Astrocyte is the ubiquitous glial cell in the brain that regulates water transport and blood flow in the brain. In addition, various neurotrophic molecules, such as glial cell line‐derived neurotrophic factor (GDNF) and brain‐derived neurotrophic factor (BDNF), are also produced, which are particularly important for the development and survival of DA neurons.^20^ Furthermore, BDNF is also essential for neuronal development, survival, and synaptic plasticity.^21^ Lipocalin 2 (LCN2) is considered to be a chemokine inducer of astrocytes and a classical pro‐inflammatory activation autocrine promoter.^22^ Compared with the A2 phenotype, which polarizes to protect neurons during hypoxia, A1‐phenotype astrocytes not only lose the ability to promote neuronal survival, growth, synaptic formation, and phagocytosis but also directly kill DA neurons by secreting neurotoxic complement 3d (C3d).^23^, ^24^

Neuroinflammation is initially a protective response of the brain. It is regulated by immune cells, cytokines, and chemokines. However, excessive neuroinflammation is toxic and could inhibit the regeneration of DA neurons.^25^ Lipopolysaccharide (LPS), as a bacterial endotoxin and glial cell activator, not only promoted the aggregation of DA neuronal loss, but also stimulated the activation and release of ROS by glial cells to mediate neuroinflammation.^26^, ^27^, ^28^ Microglia is the resident immune cell in CNS. Normally, microglia play a key role in maintaining tissue homeostasis and promoting brain development, while activated microglia and subsequent release of pro‐inflammatory factors are closely related to death of DA neurons in the brain of PD patients.^29^ The activated microglia consist of two groups of cells with different or even opposite functions.^30^ The designation of microglia anti/pro‐inflammatory status was originally developed according to the two main differentiation subtypes of T cells (Th1/Th2), based on which activated microglia could form more anti/pro‐inflammatory phenotypes, referred to as M1 or M2, respectively.^23^ Among them, M1 is a classically activated pro‐inflammatory phenotype, secreting pro‐inflammatory factors, such as tumor necrosis factor‐α (TNF‐α) and interleukin‐1β (IL‐1β). The accumulation of a large number of pro‐inflammatory factors would increase the damage of DA neurons. M2 is an alternately activated anti‐inflammatory phenotype, secreting anti‐inflammatory‐related factors, such as interleukin‐10 (IL‐10) and arginase‐1 (Arg1). The production of anti‐inflammatory factors could reduce the damage of DA neurons.^31^

To sum up, oxidative stress and neuroinflammation play a crucial role in the pathogenesis of PD, and inducible factors of these two events are also widely present in daily life. Our previous studies revealed that peripheral inflammatory stimulation induced by LPS and neurotoxicity caused by ROT co‐aggravated the damage of DA neurons.^32^ However, it is still not clear how oxidative stress and neuroinflammation interacted and participated in the development of PD. Therefore, this study intended to use ROT and LPS with sequential administration in different ways to induce PD rat model and further explored the potential synergistic effects of ROT combined with LPS on the occurrence and development of PD.

## 2 MATERIALS AND METHODS

### 2.1 Reagents

ROT and LPS (055:B5) were obtained from Sigma Chemical. Lysis Buffer and the enhanced chemiluminescence (ECL) reagent were purchased from Beyotime Institute of Biotechnology. Anti‐tyrosine hydroxylase (TH; Catalog No. ab113; sheep polyclonal), Nrf2 (Catalog No. ab31163; rabbit polyclonal), GDNF (Catalog No. ab176564; rabbit monoclonal), Ionized calcium‐binding adaptor molecule‐1 (Iba‐1; Catalog No. ab178847; rabbit monoclonal), and HO‐1 (Catalog No. ab68477; rabbit monoclonal) antibodies were bought from Abcam. IL‐1β (Catalog No. 16806‐1‐AP; rabbit polyclonal), TNF‐α (Catalog No. 17590‐1‐AP; rabbit polyclonal), IL‐10 (Catalog No. 20850‐1‐AP; rabbit polyclonal), Arg1 (Catalog No. 16001‐1‐AP; rabbit polyclonal), glial fibrillary acidic protein (GFAP; Catalog No. 16825‐1‐AP; rabbit polyclonal), BDNF (Catalog No. 28205‐1‐AP; rabbit polyclonal), Keap1 (Catalog No. 10503‐2‐AP; rabbit polyclonal), proliferating cell nuclear antigen (PCNA; Catalog No. 10205‐2‐AP; rabbit polyclonal), NDUFS3 (Catalog No. 15066‐1‐AP; rabbit polyclonal), succinate dehydrogenase (SDHA; Catalog No.14865‐1‐AP; rabbit polyclonal), and β‐actin antibodies were bought from Proteintech Group. NQO1 (Catalog No. ER1802‐85; rabbit polyclonal) and LCN2 (Catalog No. ET1703‐39; rabbit monoclonal) antibodies were from Huabio. C3d (Catalog No. AF2655‐SP; Goat Polyclonal) was bought from R&D Systems. SYBR green polymerase chain reaction (PCR) master mix was purchased from Bio‐Rad. RNAiso plus was from Takara Biotech Co., Ltd.

### 2.2 Animals and treatment

**FIGURE 1:**
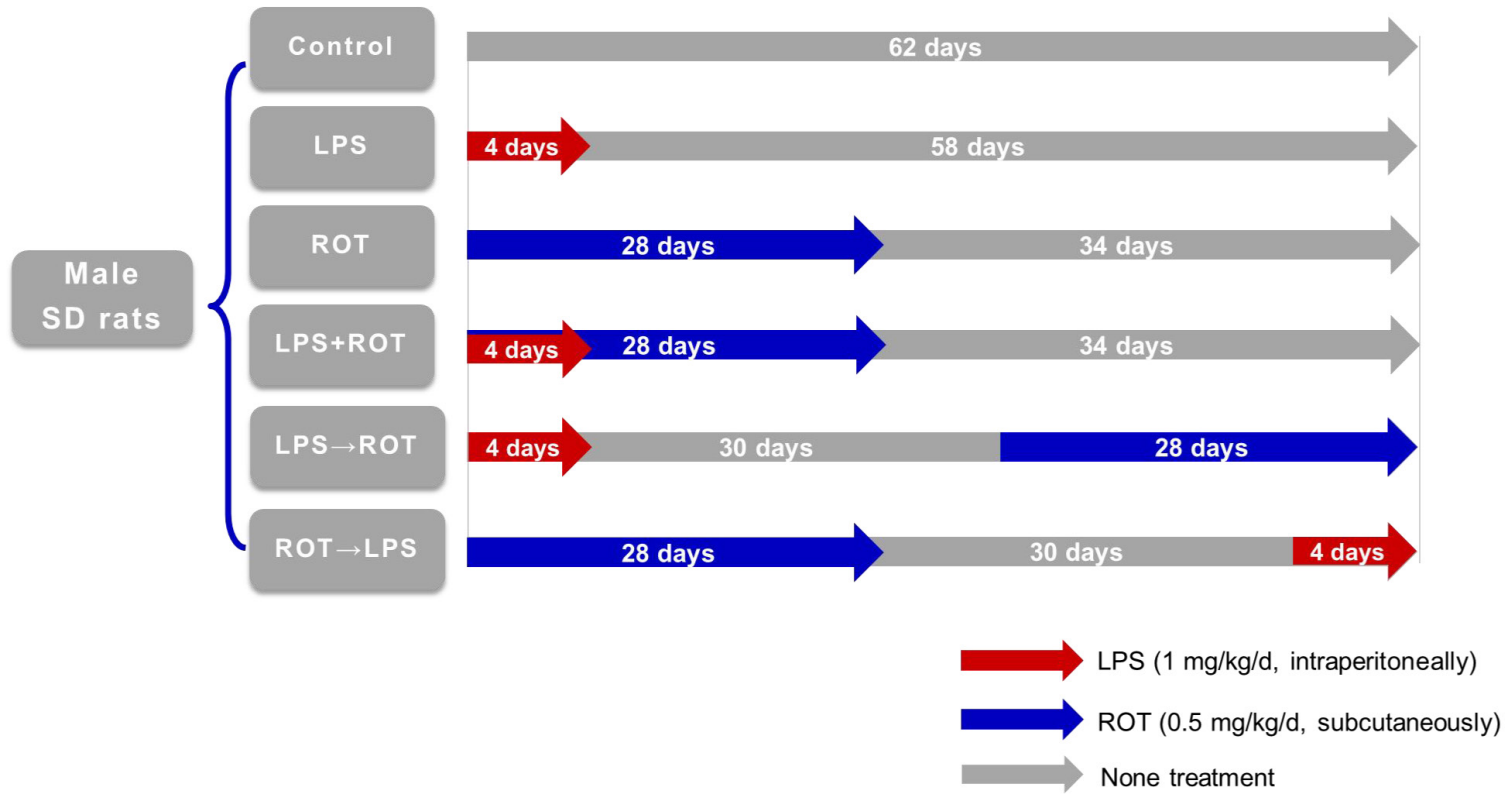
Grouping and time schedule of LPS and ROT administration.

Adult male Sprague–Dawley rats (220–260 g) were purchased from the Liaoning Long Life Biotechnology Co., Ltd. LPS‐ or ROT‐induced rat PD model was applied in this study.^33^, ^34^ All animal experiments were performed in accordance with Chinese Guidelines of Animal Care and Welfare and the present study was approved by the Animal Care and Use Committee of Zunyi Medical University. All animals were housed in a temperature (19–25°C) and humidity (40%–70%) environment and given access to food and water ad libitum. The experimental animals were randomly divided into 6 groups: control, LPS (1 mg/kg/d), ROT (0.5 mg/kg/d), LPS + ROT (simultaneous administration), LPS→ROT, and ROT→LPS (sequent administration) groups. LPS (1 mg/kg/d) was injected intraperitoneally for 4 consecutive days. ROT (0.5 mg/kg/d) was subcutaneously injected six times a week for consecutive 4 weeks. The detail of specific grouping method is shown in Figure 1. After the end of the administration, behavioral tests were performed uniformly and then animals were sacrificed.

### 2.3 Rotarod test

Rotarod test is a common test used to evaluate the effects of drugs on animal behavior dysfunction. Before the experiment, all the rats were trained on the rotating rod until they remained on the rod for at least the specified time. The starting speed of the test was 10 rpm, increasing by 5 rpm every 30 s until rats fell off the rotating rod. The behavior changes in rats were analyzed by recording the time that each rat stayed on the rod.

### 2.4 Open field test

Open field test is a sensorimotor test used to determine the general activity level, total motor activity and exploratory habits of rodent models of neurological disorders. In this study, each rat was placed in a separate open field area, and rat behavioral parameters were recorded within 10 min. Before each round of test, the equipment was cleaned with 75% alcohol solution to avoid odor interference. After the experiment, the total moving distance of rats was calculated.

### 2.5 Tissue preparation and immunofluorescence staining

After the behavioral tests, anesthetized rats were perfused with PBS before fixation with 4% paraformaldehyde. The brain was peeled off and fixed with 4% paraformaldehyde for 1 week. Within dehydration and embedding into paraffin blocks for subsequent dyeing, the wax block was placed on a paraffin microtome (Leica) and the brain was cut into a six microns cross‐section attached to a glass slide. Brain slices were separately treated with 3% hydrogen peroxide and 0.1 M citrate buffer and blocked with goat serum. These slices were incubated overnight at 4°C with the following primary antibodies: TH (1:500, the marker of DA neurons) and TH (1:100) + NDUFS3 (1:100). Then, the corresponding fluorescent secondary antibody was incubated for 30 min at 37°C. The images of TH‐positive neurons in SN and TH‐positive neurons containing NDUFS3 were presented by Olympus microscope and the quantification of DA neurons were finally counted and averaged.

### 2.6 Immunohistochemical staining

The slices attached to the brain tissue were dried and treated with 3% hydrogen peroxide and 0.1 M citrate buffer, and sealed with goat serum in an oven at 37°C. The slices were incubated overnight at 4°C with the following primary antibodies: GFAP (1:300, the marker of astrocytes) and Iba‐1 (1:200, the marker of microglia). Brain sections were incubated with secondary antibody working solution at 37°C for 20 min followed by incubation with biotin for 18 min and developed with DAB developer. Five rats in each group were used for detection, and 2–3 brain slices were taken from each rat for measurement and the mean value was calculated. The images of GFAP‐positive astrocytes and Iba1‐positive microglia were presented by an Olympus microscope, and then the density was analyzed by ImageJ software and the average value was taken.

### 2.7 Western blot analysis

The total protein was extracted from rat midbrain tissue with lysate containing protease inhibitor. Nucleoplasmic protein extraction kit (Solabio) was used to extract nuclear and cytoplasmic parts. The protein concentrations were quantified by a BCA kit. An equal amount of protein was separated by a 10% Bis‐Tris Nu‐PAGE gel and transferred to a polyvinylidene fluoride (PVDF) membrane. Then, membranes were blocked with 7% skimmed milk for 3 h and incubated overnight in the primary antibody at 4°C. The primary antibodies were TH (1:2000), Iba‐1 (1:800), TNF‐α (1:1000), IL‐1β (1:1000), IL‐10 (1:1000), Arg1 (1:1000), GFAP (1:2000), LCN2 (1:1000), C3d (1:1000), GDNF(1:1000), BDNF(1:2000), Nrf2 (1: 1000), HO‐1 (1:1000), NQO1 (1:1000), Keap1 (1:2000), PCNA (1:2000), NDUFS3 (1:1000), SDHA (1:1000), and β‐actin (1:2000). Subsequently, membranes were incubated for 1 h with a horseradish‐peroxidase‐conjugated anti‐mouse IgG antibody or anti‐rabbit IgG at 1:2000 dilution, and the ECL substrate was detected. Quantitative analysis (Bio‐Rad) software was used for statistical analysis of the results.

### 2.8 Real‐time RT‐PCR assay

Total RNA was extracted with Trizol reagent and purified by RNeasy kit. Nrf2, NQO1, Keap1, HO1, and β‐actin genes were amplified using forward and reverse primers. SYBR Green Supermix was used to perform real‐time RT‐PCR in accordance with the instructions, and then the CFX96 real‐time RT‐PCR detection system (Bio‐Rad) was applied. The target gene expression level was normalized withβ‐actin expression level. The control group was set to 100%.

### 2.9 Malondialdehyde (MDA) and SOD assays

The content of MDA was determined with a lipid peroxidation MDA detection kit (Beyotime) and calculated with a standard curve according to the manufacturer's instructions in kit. Total SOD detection kit WST‐8 (Beyotime) was used to detect SOD activity and the calculation was performed according to the manufacturer's instructions. All experiments were repeated three times independently.

### 2.10 Statistical analysis

Data were presented as mean ± SEM. The control group was set as 100% and the differences between the other groups and control group were compared. Statistical comparison used SPSS statistical software for one‐way analysis of variance (ANOVA) and normal distribution test. When analysis of variance showed significant differences, all pairwise comparisons among means were accessed by Bonferroni's post hoc *test* with correction. All data were tested for normality. Data that did not exhibit a normal/Gaussian distribution were analyzed by non‐parametric equivalents. A value of *p* < 0.05 was considered statistically significant.

## 3 RESULTS

### 3.1 Effects of different sequential administration of LPS and ROT on motor dysfunction and DA neuronal damage in rats

**FIGURE 2:**
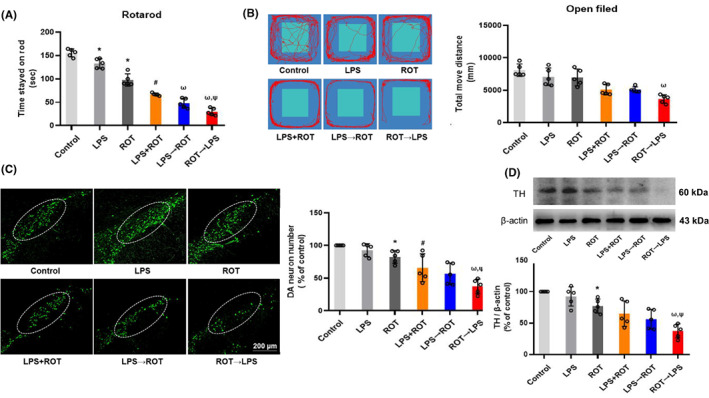
Effects of different sequential administration of LPS and ROT on motor dysfunction and DA neuronal damage in rats. Rotating rod test (A) and open field test (B) were performed on rats after LPS and ROT administration. (C) TH‐positive neurons number was counted after rat midbrain sections immunofluorescence staining (Scale bar = 200 μm). (D) TH protein expression was detected by Western blot assay. Data were represented as mean ± SEM from five rats. *p < 0.05 compared with control group, ^#^ *p* < 0.05 compared with LPS group, ^ω^ *p* < 0.05 compared with LPS + ROT, ^Ψ^ *p* < 0.05 compared with LPS→ROT group.

To investigate whether LPS and ROT had initiating or promoting factors in the pathogenesis of PD, effects of different sequential administration of LPS and ROT on rat behavior changes and DA neuronal damage were observed. First, as shown in Figure 2A, the time of rats stayed on rod in LPS alone and ROT alone groups was shorter than that in control group. Compared with LPS group, time of rats stayed on rod in LPS + ROT group was shorter than that in LPS group. In addition, compared with LPS + ROT group, time of rats stayed on rod in ROT→LPS and LPS→ROT groups were shortened, in which time in ROT→LPS group was much shorter than that in LPS→ROT group. However, in open field test (Figure 2B), an obvious decrease was just in ROT→LPS group.

To further investigate the effects of different sequential administration of LPS and ROT on DA neuronal loss in rats, the quantification of TH‐positive DA neurons in rat SN and the expression of TH protein were detected. As shown in Figure 2C, compared with single LPS or ROT group, the number of DA neurons in rat midbrain of LPS + ROT group was reduced. Compared with LPS + ROT group, DA neuronal number in ROT→LPS group was apparently decreased, whereas no significant difference of DA neurons between LPS + ROT and LPS→ROT groups was discerned. Similar results were exhibited in TH protein expression detection (Figure 2D, Figure S1). These results indicated that ROT and LPS synergistically aggravated DA neuronal damage of rats, and the later application of LPS could more effectively promote the damage of ROT to DA neurons.

### 3.2 Effects of different sequential administration of LPS and ROT on mitochondrial respiratory chain of DA neurons in rats

**FIGURE 3:**
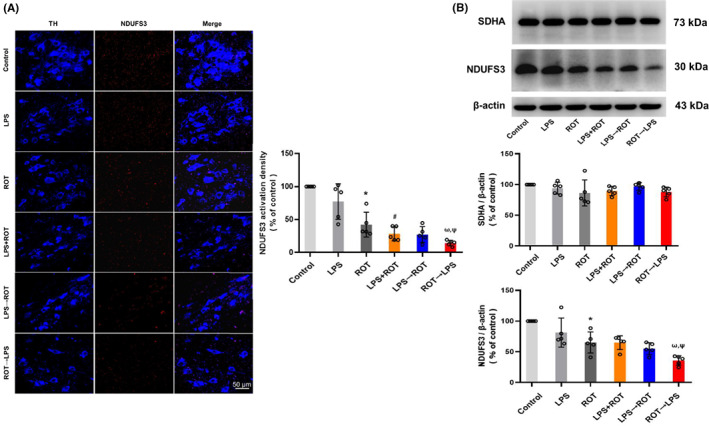
Effects of different sequential administration of LPS and ROT on the mitochondrial respiratory chain of DA neurons in rat midbrain. (A) The density of mitochondrial complex I NDUFS3 in rat midbrain was recorded via immunofluorescence staining (Scale bar = 50 μm). (B) NDUFS3 and SDHA protein expressions in rat midbrain were tested by Western blot assay. Data were represented as mean ± SEM from five rats. *p < 0.05 compared with control group, ^#^ *p* < 0.05 compared with LPS group, ^ω^ *p* < 0.05 compared with LPS + ROT, ^Ψ^ *p* < 0.05 compared with PS→ROT group.

Next, we verified the effects of different sequential administration of LPS and ROT on the mitochondrial respiratory chain of DA neurons in rat SN. First, TH and NDUFS3 protein expressions in rat midbrain were double‐stained by double‐labeled immunofluorescence. Compared with control group, the expression of NDUFS3 protein in ROT group was decreased. Compared with the single LPS or ROT administration group, the expression of NDUFS3 protein in LPS + ROT group was down‐regulated. Furthermore, compared with LPS + ROT group, NDUFS3 protein expression in ROT→LPS group was reduced, while no significant difference was shown in LPS→ROT group (Figure 3A). In addition, the protein expression levels of NDUFS3 and SDHA in rat midbrain were determined. Similar phenomenon was discerned in NDUFS3 protein detection but not in SDHA (Figure 3B, Figure S1).

### 3.3 Effects of different sequential administration of LPS and ROT on microglia activation and secretion of cytokines in rat midbrain

**FIGURE 4:**
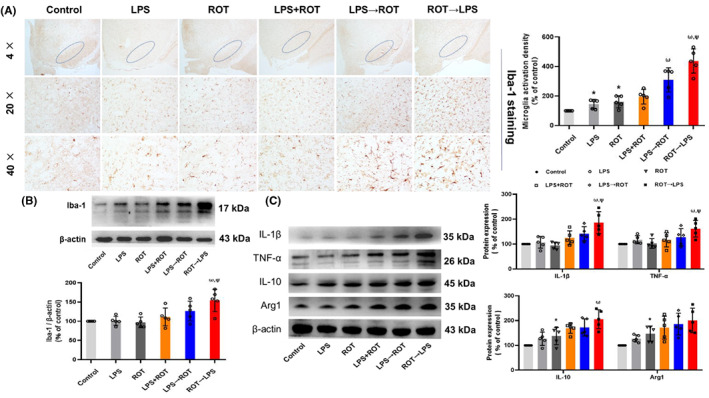
Effects of different sequential administration of LPS and ROT on microglia activation and secretion of cytokines in rat midbrain. (A)The activated microglia in the substantia nigra of rat midbrain were detected by immunocytochemical staining with anti‐Iba‐1 antibody. The density of activated microglia was recorded. (B) Iba‐1 protein expression was tested by Western blot assay. (C) IL‐1β, TNF‐α, IL‐10, and Arg1 protein expressions was determined by Western blotting. Data were represented as mean ± SEM from 5 rats. *p < 0.05 compared with control group, ^#^ *p* < 0.05 compared with LPS group, ^△^ *p* < 0.05 compared with ROT group, ^ω^ *p* < 0.05 compared with LPS + ROT group, ^Ψ^ *p* < 0.05 compared with LPS→ROT group.

Since glial cells were involved in neuroinflammatory response, microglia activation and the production of various cytokines were assessed. As shown in Figure 4A of Iba‐1 immunohistochemical staining analysis, compared with control group, single LPS‐ or ROT‐induced microglia activation. However, no obvious difference of microglia activation between LPS /ROT and LPS + ROT groups was indicated. Furthermore, compared with LPS + ROT group, apparent microglia activation was demonstrated in ROT→LPS and LPS→ROT groups, where ROT→LPS induced more microglia activation than LPS→ROT. In addition, similar results were present in Iba‐1 protein level detection (Figure 4B, Figure S1).

Furtherly, the protein levels of M1‐type microglia pro‐inflammatory factors, such as IL‐1β and TNF‐α, and M2‐type microglia anti‐inflammatory factors, such as IL‐10 and Arg1, were tested. As shown in Figure 4C and Figure S1, the increase in pro‐inflammatory factors (IL‐1β and TNF‐α) in ROT→LPS group was the most obvious. However, there was no significant difference in increase of anti‐inflammatory factors (IL‐10 and Arg1) among different groups. These results indicated that ROT combined with LPS to aggravate microglia activation and pro‐inflammatory cytokine secretion, and after ROT destroyed the redox balance, LPS provided inflammatory stimulation to further promote microglia activation and pro‐inflammatory cytokine secretion.

### 3.4 Effects of different sequential administration of LPS and ROT on astrocytes activation and release of cytokines in rat midbrain

**FIGURE 5:**
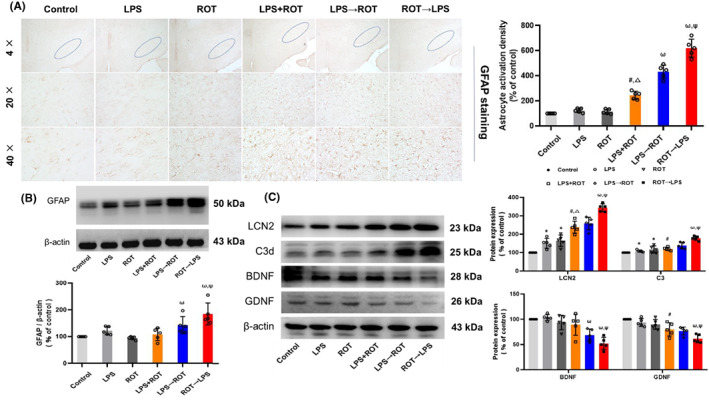
Effects of different sequential administration of LPS and ROT on activation and cytokines release of astrocytes in rat midbrain. (A)The activated astrocytes in the substantia nigra of rat midbrain were assessed by immunocytochemical staining with anti‐GFAP antibody. The density of activated astrocytes was measured. (B) GFAP protein expression was detected by Western blotting. (C) LCN2, C3, BDNF, and GDNF protein expressions were tested by Western blot assay. Data were represented as mean ± SEM from 5 rats. *p < 0.05 compared with control group, ^#^ *p* < 0.05 compared with LPS group, ^△^ *p* < 0.05 compared with ROT group, ^ω^ *p* < 0.05 compared with LPS + ROT group, ^Ψ^ *p* < 0.05 compared with LPS→ROT group.

In addition to microglia, astrocytes participated in DA neurodegeneration. Immunohistochemical staining analysis (Figure 5A) showed that, compared with control group, single LPS or ROT did not induce the activation of astrocytes but LPS + ROT elicited astrocytes activation. Compared with LPS + ROT group, both LPS→ROT and ROT→LPS administration induced obvious astrocytes activation, where ROT→LPS induced more astrocytes activation than LPS→ROT. GFAP protein level measurement (Figure 5B, Figure S1) was consistent with results of immunohistochemical staining. Moreover, compared with LPS→ROT group, ROT→LPS caused more A1‐type astrocytes neurotoxic factors, such as LCN2 and C3 release, and less A2‐type astrocytes neurotrophic factors, such as BDNF and GDNF release (Figure 5C, Figure S1).

### 3.5 Effects of different sequences of administration of LPS and ROT on brain oxidation balance and activation of Nrf2 signaling in rat midbrain

**FIGURE 6:**
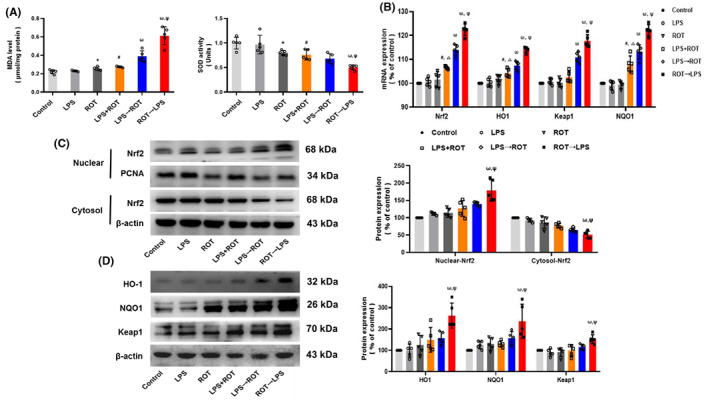
Effects of different sequential administration of LPS and ROT on the balance of oxidation and activation of Nrf2 signaling in the midbrain of rats. (A) The levels of MDA and SOD in rat midbrain were detected. (B) The mRNA expressions of Nrf2, HO‐1, NQO1, and Keap1 in rat midbrain were measured by real‐time RT‐PCR. (C) Nrf2 protein expression in cytosol and nuclear was tested by Western blot assay, respectively. (D) HO‐1, NQO1 and Keap1 protein expressions were detected by Western blotting. Data were represented as mean ± SEM from five rats. *p < 0.05 compared with control group, ^#^ *p* < 0.05 compared with LPS group, ^ω^ *p* < 0.05 compared with LPS + ROT group, ^Ψ^ *p* < 0.05 compared with LPS→ROT group.

From the above results, we found that ROT→LPS caused more DA neuronal damage than LPS→ROT. It is well‐known that ROT is closely associated with redox balance. Next, the effects of different sequences of administration of LPS and ROT on brain oxidation balance and activation of Nrf2 signaling in rat midbrain were explored. First, as shown in Figure 6A, MDA and SOD levels detection indicated that compared with control group, LPS + ROT and ROT alone not LPS alone caused increase in MDA and decrease in SOD. Compared with LPS + ROT group, more increase in MDA and more decrease of SOD were displayed in ROT→LPS group than those in LPS→ROT group. Second, activation of Nrf2 signaling in rat midbrain was analyzed. As shown in Figure 6B, compared with LPS or ROT group, LPS + ROT‐induced mRNA expressions of Nrf2, HO‐1, Keap1, and NQO1. Compared with LPS + ROT group, ROT→LPS induced more expressions of these genes than LPS→ROT. Consistent with real‐time RT‐PCR analysis, compared with LPS→ROT group, ROT→LPS caused more cytosol Nrf2 to enter nucleus (Figure 6C, Figure S1) and also more protein expressions of HO‐1, Keap1, and NQO1 (Figure 6D, Figure S1).

## 4 DISCUSSION

In the present study, the changes in motor behavior, DA neurons quantification, and their mitochondrial respiratory chain, glial cells activation and secreted cytokines, Nrf2 pathway, and redox balance in the brain of rats were evaluated. It was found that LPS‐induced neuroinflammation and ROT‐induced oxidative stress synergistically aggravated motor dysfunction, DA neuron damage, activation of glial cells and release of related mediators, activation of Nrf2 signaling, and destruction of oxidative balance. In addition, further studies indicated that after ROT‐induced oxidative stress caused direct damage to DA neurons, LPS‐induced inflammatory effects had stronger promoting effects on the above aspects. Together, this study demonstrated that oxidative stress played a certain role in initiating action, and neuroinflammation could enhance the promoting role of oxidative stress in the pathogenesis of PD.

The characteristic motor symptoms of PD are mainly bradykinesia and static tremor. Meanwhile, current evidence proved that PD could present anxiety‐like behavior.^35^ For PD animal models, the change in motor ability in rats is usually evaluated by rotating rod test, and the appearance of exploration and anxiety‐like behavior in rats is observed by open field test. In this study, ROT‐induced oxidative stress combined with LPS‐induced neuroinflammation to aggravate rat motor dysfunction, and the later induction of LPS could more effectively promote ROT‐induced damage to motor function. It is worth noting that LPS/ROT‐induced motor dysfunction was mainly manifested in motor coordination ability, but no significant effects on the autonomous exploration and anxiety‐like behavior were shown. Furthermore, the progressive loss of DA neurons in SN is the major feature of PD, and the cellular mitochondrial complex I NDUFS3 was introduced into the mitochondria after nuclear transcription, and this process was down‐regulated in dysfunctional mitochondria.^9^ The present study found that the expression of NDUFS3 was decreased along with the aggravation of midbrain DA neuron injury. Since the pathogenesis of PD is very complex and has not yet been clarified, oxidative stress and neuroinflammation, as the important factors that induce PD development, have attracted extensive attention. ROT is a highly lipopilic pesticide, which plays its function as a mitochondrial complex I inhibitor in brain after long‐term exposure, including damaging the normal function of DA neurons, increasing the production of ROS, causing imbalance of redox in brain, and further inducing oxidative stress damage to neurons.^36^ ROT is a typical example of exogenous toxins simulating the clinical and pathological characteristics of PD in animal models and is widely used in the construction of PD animal model to explore the therapeutic effects of drugs on the oxidative stress pathway of PD.^37^, ^38^, ^39^ LPS is an endotoxin of gram‐negative bacteria, which is widely used in investigating the role of the inflammatory process in PD and the anti‐inflammatory treatment of PD.^40^, ^41^, ^42^ Therefore, this study induced oxidative stress and inflammatory state in rat brain by introducing ROT and LPS, respectively. Furthermore, M2‐type microglia could play an important role in brain environment monitoring, cell maintenance and innate immunity. Alternately, activated M1‐type microglia release a large number of neurotoxic and pro‐inflammatory factors to damage DA neurons, which in turn damaged DA neurons generated neurotoxic factors, such as damage‐associated molecular patterns (DAMPs), further resulting in microglia activation and forming a self‐amplification cycle of neuronal damage and microglia activation and thus leading to more DA neuronal death.^43^ Studies have shown that inhibiting the polarization of microglia to M1 phenotype could alleviate neuroinflammation.^44^, ^45^ However, this study found that with the aggravation of ROT combined with LPS‐caused damage, the secretion of anti‐inflammatory factors of microglia in the midbrain of rats was increased similar to that of pro‐inflammatory factors, indicating that the activation states of microglia were more dynamic and varied between the two extreme states of M1 and M2‐type microglia.^46^ Although the mechanisms of oxidative stress‐ and neuroinflammation‐induced PD were different, it was not comprehensive to discuss these two events separately. Recent studies revealed that over‐structured ROS promoted the polarization of microglia to M1 phenotype.^47^, ^48^, ^49^, ^50^ Here, the present study indicated that ROT‐induced oxidative stress combined with LPS‐induced neuroinflammation could aggravate the damage of DA neurons and mitochondrial dysfunction, the activation of microglia and the secretion of related cytokines. Furtherly, after ROT caused direct oxidative damage to DA neurons, LPS exerted inflammatory effects, which promoted the above aspects more seriously. These results suggested that oxidative stress played an initial role in the PD process to a certain extent compared to neuroinflammation, while neuroinflammation strengthened the role of oxidative stress on the progression of PD. In addition, the late treatment of LPS resulted in a higher proportion of M1‐type pro‐inflammatory factor secretion in the brain of rats, at which time the inflammatory effect was in its full state, which may also be the reason for the more serious damage of DA neurons in the brain of rats.

To further confirm the initiation of oxidative stress in PD development, the effects of LPS and ROT sequential administration on brain oxidation condition and Nrf2 signaling were assessed. MDA, one of the most commonly used indicators of lipid peroxidation, and SOD, which could counter the destructive reaction of superoxide, are widely used determine the redox state in brain.^51^, ^52^ This study found that ROT and LPS synergistically increased MDA level, especially first stimulation of ROT followed by LPS‐induced apparent increase in MDA level, while SOD level detection were opposite. In addition, as a major regulator of oxidative stress, Nrf2 could regulate the redox state of cells in response to oxidative stress.^53^ Normally, Nrf2 is located in the cytoplasm of DA neurons in SN.^54^ In patients with PD, Nrf2 is activated and enters the nucleus, and the expressions of NQO1 and HO‐1 in Nrf2 downstream pathway are subsequently up‐regulated.^55^ Moreover, studies have confirmed that a large number of candidate drugs can attenuate PD through the activation of Nrf2 signaling.^56^, ^57^, ^58^ In this study, it was found that ROT combined with LPS aggravated the destruction of oxidative balance state in brain compared with single LPS/ROT administration, and compared with LPS + ROT group, ROT stimulation followed by LPS could enhance Nrf2 signaling activation and further the destruction of oxidative balance condition. It was suggested that neuroinflammation could aggravate oxidative damage as a promoting factor after the brain redox state was destroyed by ROT. In the case of oxidative equilibrium destruction, activation of the antioxidant element Nrf2 was observed. It was speculated that Nrf2 was activated under oxidative stress state, although its downstream signaling had certain antioxidant effects. Furtherly, these antioxidant effects were not sufficient to reverse the damage, especially in the neuroinflammatory damage caused by following sequential administration of LPS.

At present, clinical medication for PD patients can only relieve the symptoms of PD, but cannot prevent the progression of PD, and side effects always exist in the course of medication.^59^ Therefore, it is very important to explore the progression of PD. Studies have shown that oxidative stress and neuroinflammation can play a synergistic role to accelerate the occurrence and development of PD.^32^ However, it is still unclear how this synergistic effect affects the progression of PD and whether there is any initiating factor. This study primary investigated the effects of different sequential administration of LPS and ROT on DA neuronal loss in rat midbrain. Data showed that LPS‐induced neuroinflammation and ROT‐induced oxidative stress synergistically aggravated DA neuronal loss. Furtherly, DA neuronal loss induced by ROT followed by LPS administration was more severe than that elicited by LPS followed by ROT application. Thus, this study was a pilot study just suggesting that oxidative stress followed by neuroinflammation caused more DA neuronal loss than neuroinflammation followed by oxidative stress. Despite understanding the direct role of LPS and ROT in PD pathophysiology, the mechanisms responsible for crosstalk between these two factors associated with PD etiology was far from being illuminated. Here, there are several limitations in this study. First, the time‐course study on both LPS or ROT alone administration and LPS combined with ROT application would provide more detail information. Second, why oxidative stress followed by neuroinflammation damaged more DA neurons than neuroinflammation followed by oxidative stress was unrevealed. Thus, the underlying mechanisms warrant further exploration. This study provides a baseline for understanding the crosstalk between neuroinflammation and oxidative stress on PD pathogenesis.

## 5 CONCLUSIONS

This study demonstrated that neuroinflammation combined with oxidative stress aggravated DA neuronal loss. Furtherly, oxidative stress followed by neuroinflammation caused more DA neuronal loss than neuroinflammation followed by oxidative stress.

## AUTHOR CONTRIBUTIONS

Feng Zhang conceived and designed the experiments. Jing‐Yi He participated in the experiments performance and Dai‐Di Li, Qian Wen, Ting‐Yang Qin, Hong Long, Shi‐Bin Zhang, and Feng Zhang finished data analysis. Jing‐Yi He wrote this manuscript and Feng Zhang revised the article. All authors read and approved the final manuscript.

## CONFLICT OF INTEREST STATEMENT

The authors declare that they have no conflict of interest.

## Supporting information


Figure S1

## Data Availability

Data that support the findings of this study were available from the corresponding author upon reasonable request.
