# Supplementary material for: Synergistic effects of lipopolysaccharide and rotenone on dopamine neuronal damage in rats
Source: CNS Neurosci Ther. 2023 Mar 21;29(8):2281–91. doi: 10.1111/cns.14180 (PMC10352892; doi:10.1111/cns.14180)

Full unedited gel/blot for
Figure - 2D TH

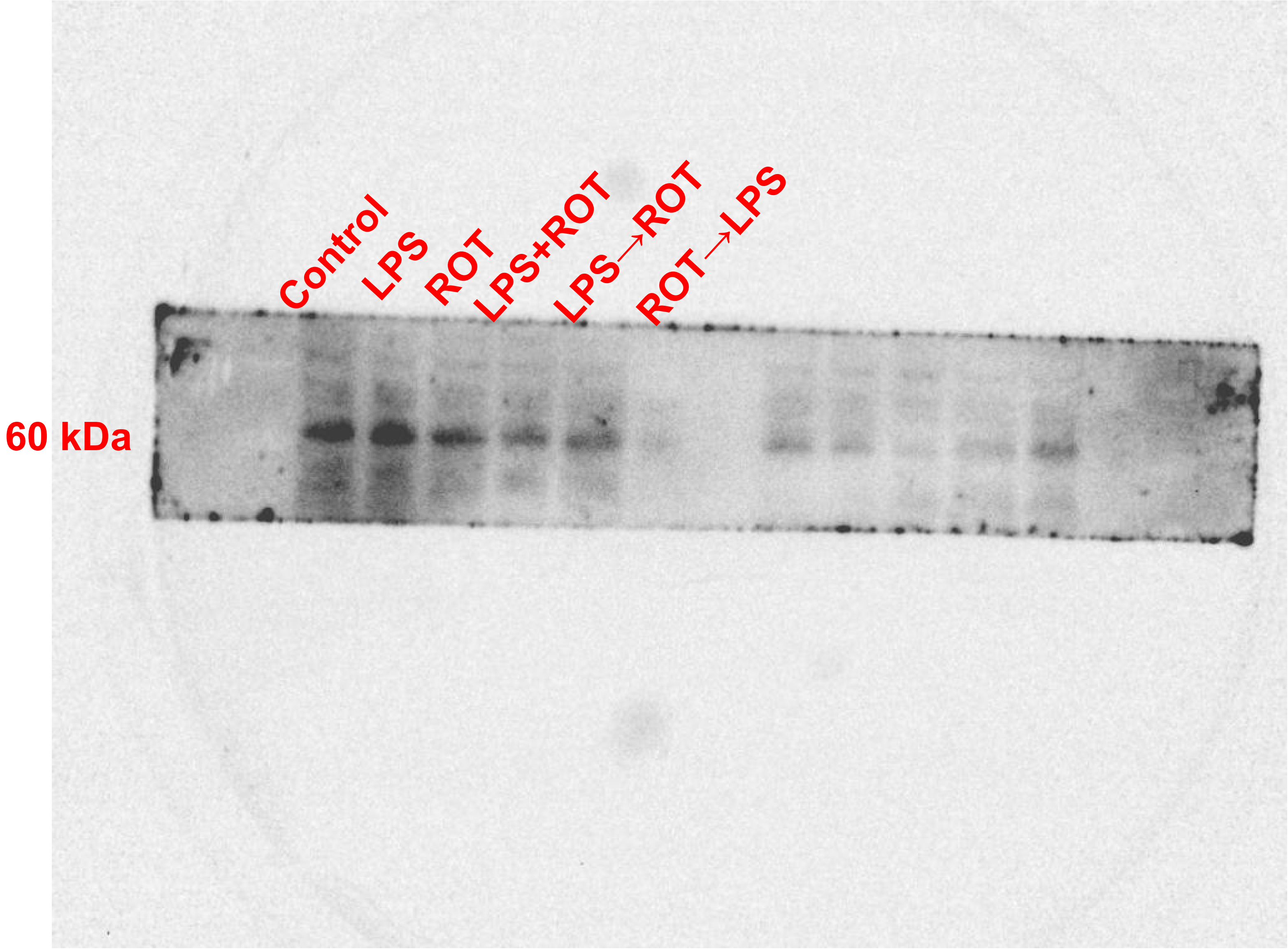

Full unedited gel/blot for
Figure - 3B SDHA

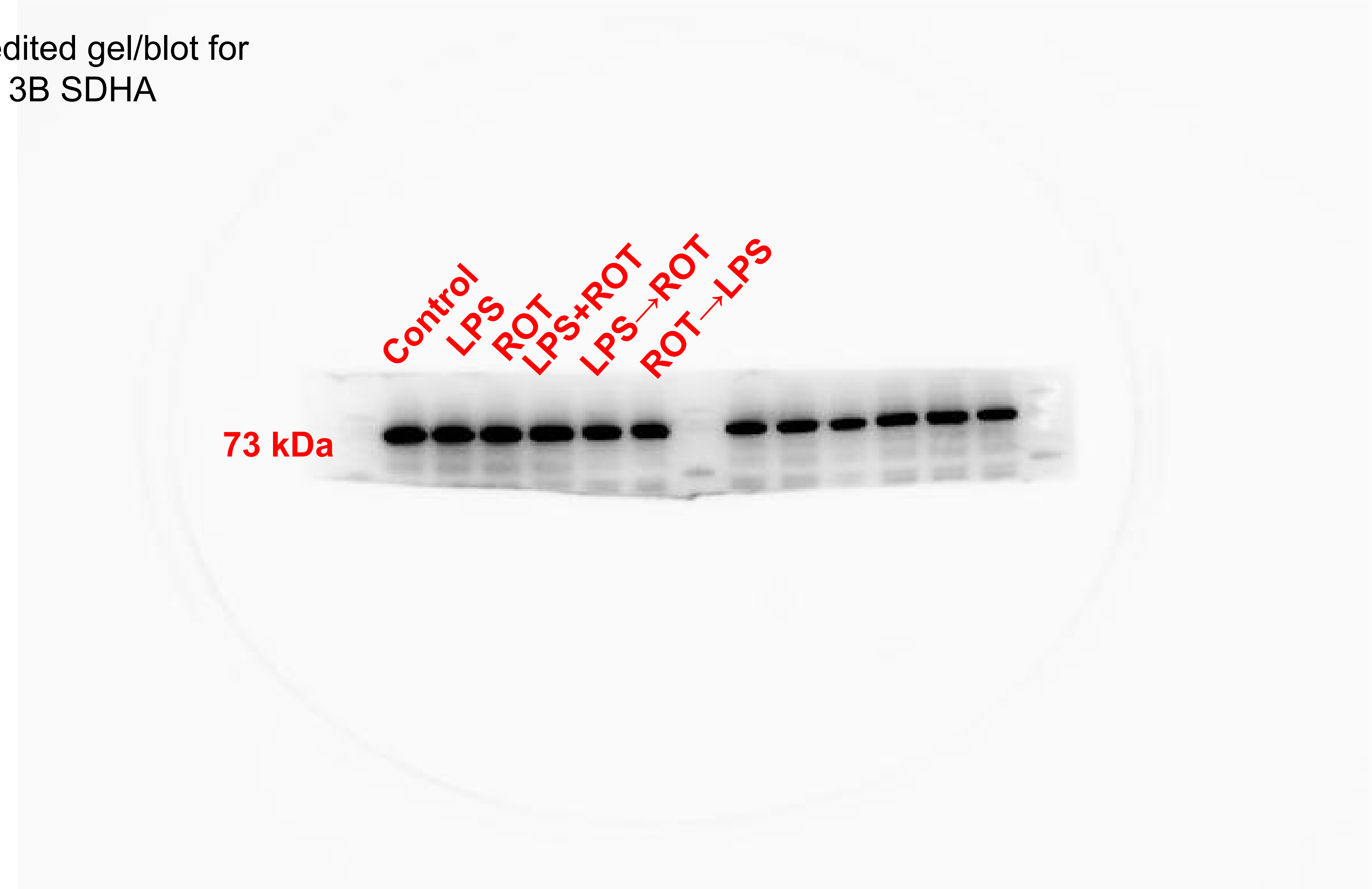

Full unedited gel/blot for
Figure - 3B NDUFS3

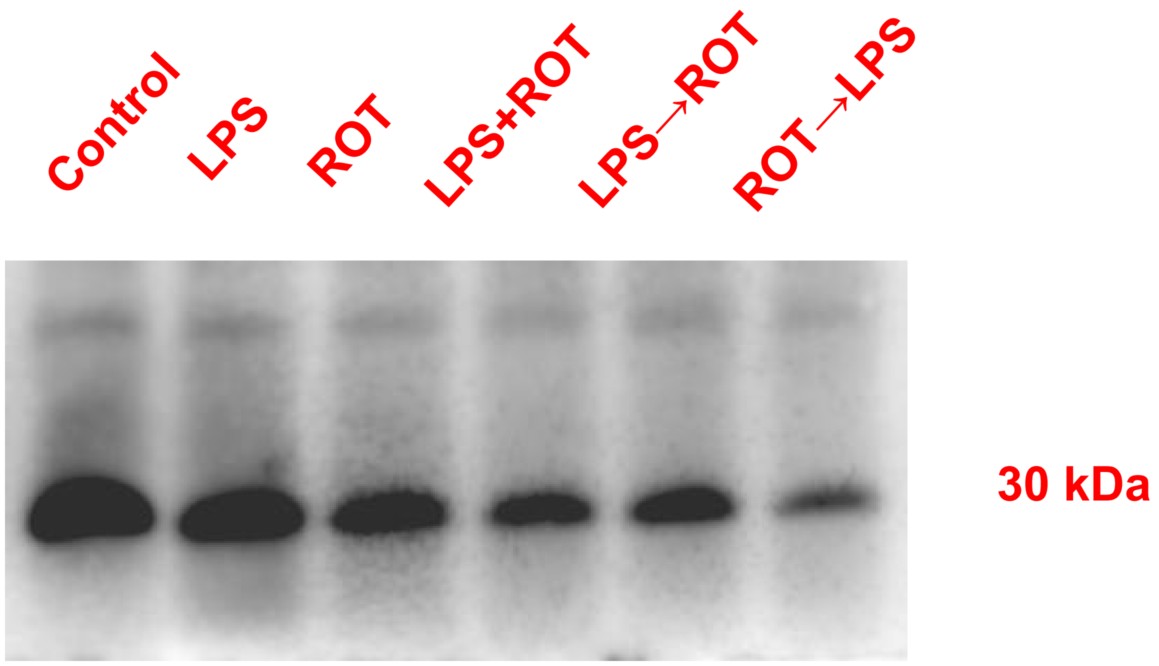

Full unedited gel/blot for
Figure - 4B Iba-1

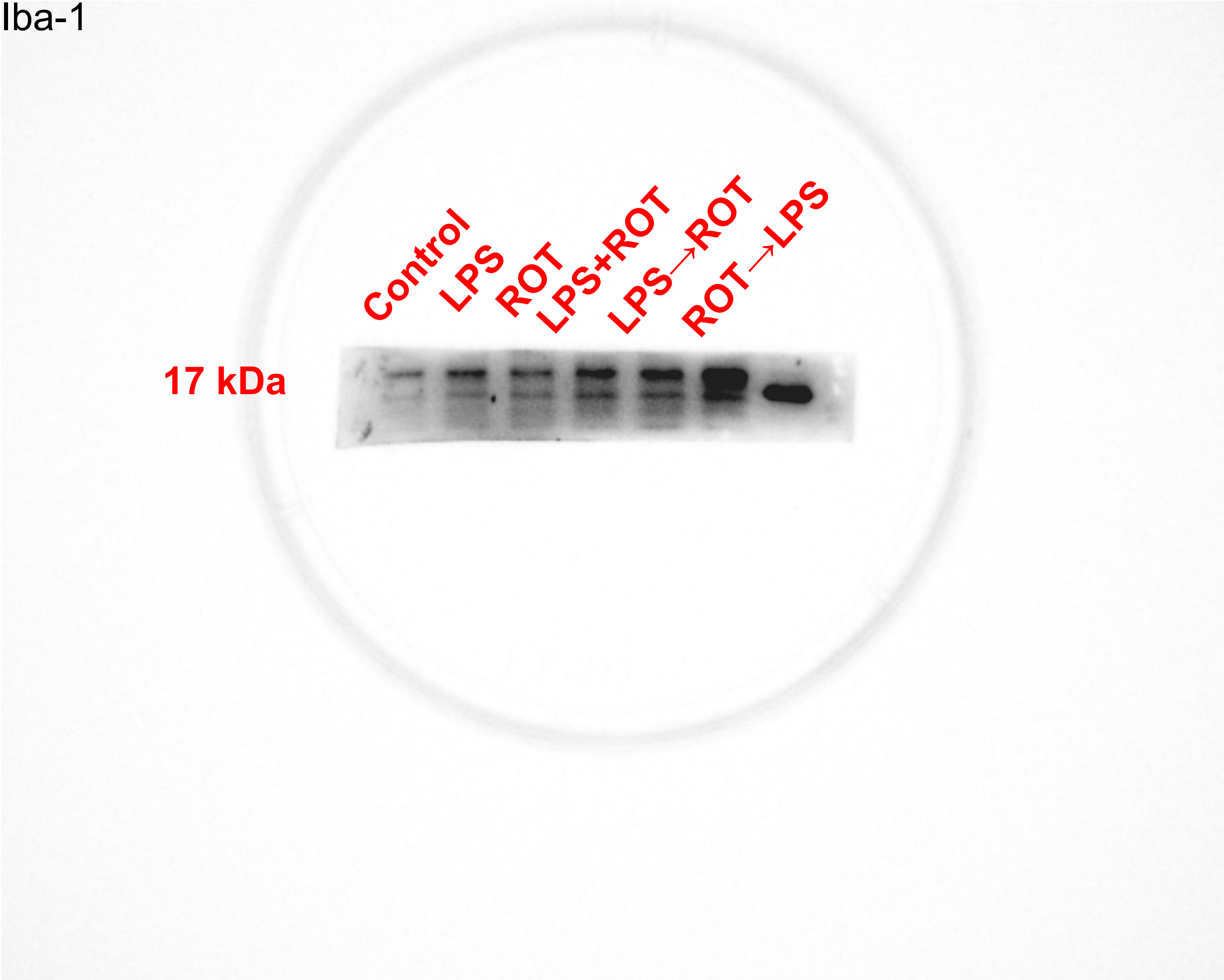

Full unedited gel/blot for
Figure - 4C TNFα

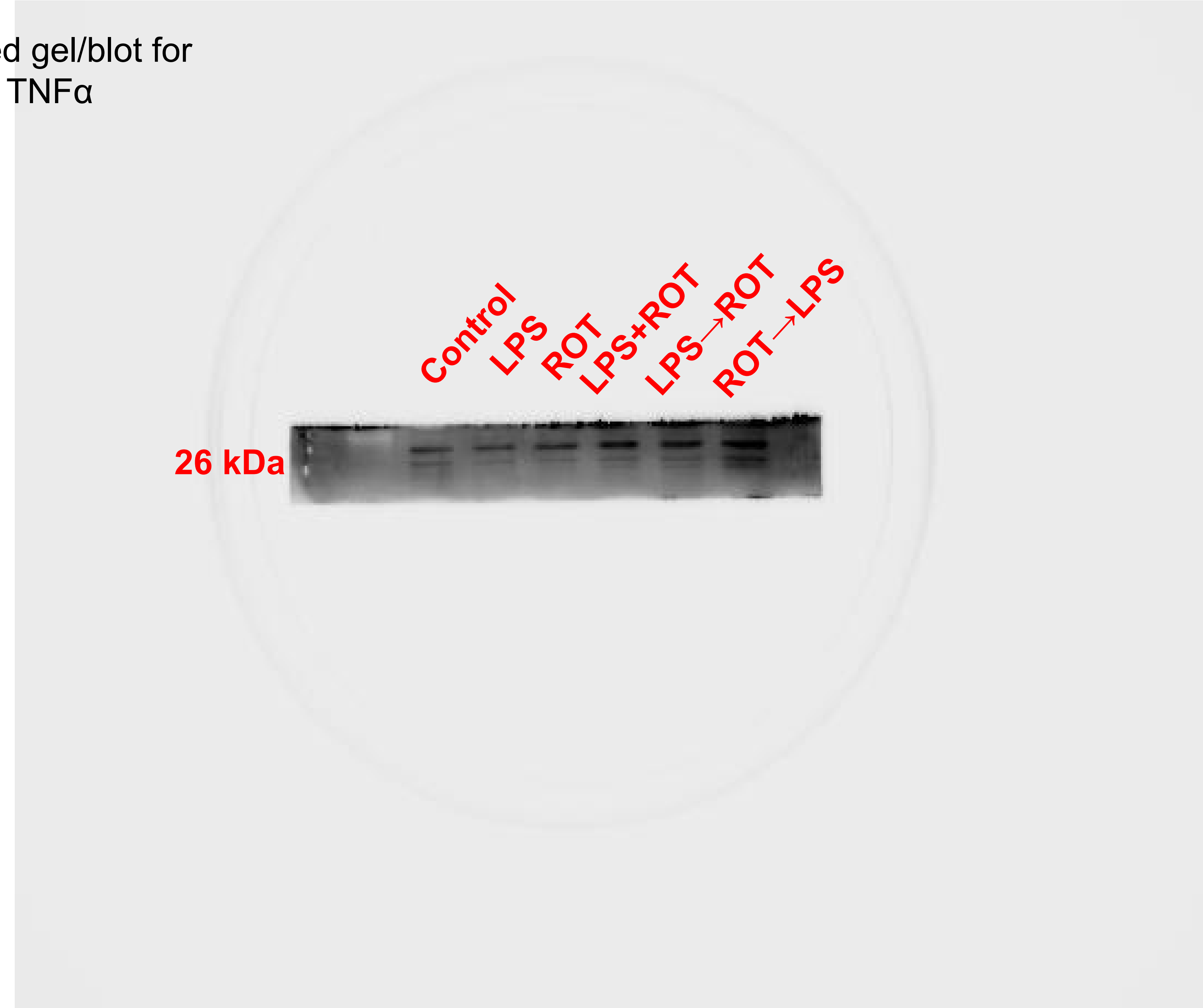

Full unedited gel/blot for
Figure - 4C IL-1β

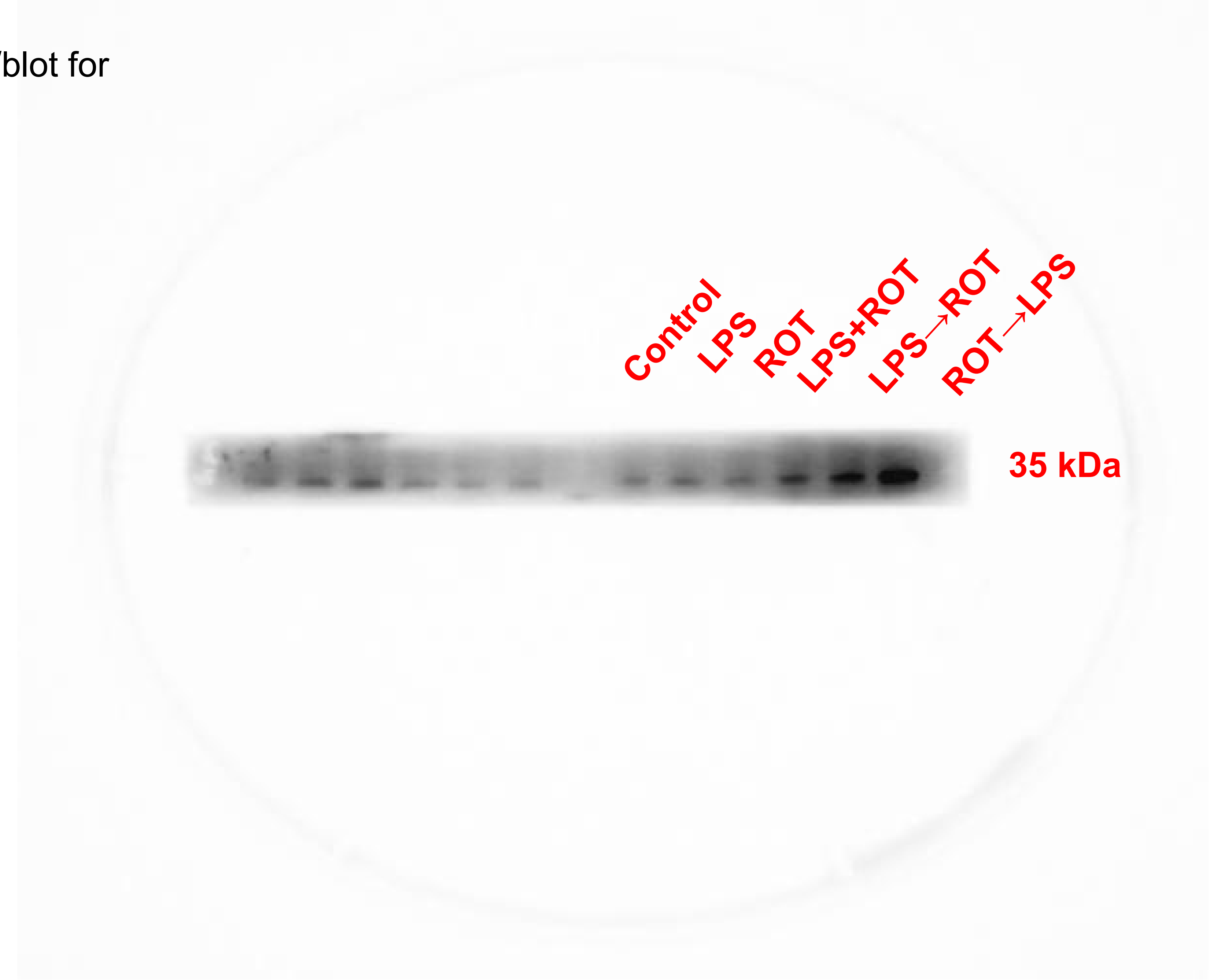

Full unedited gel/blot for
Figure - 4C IL-10

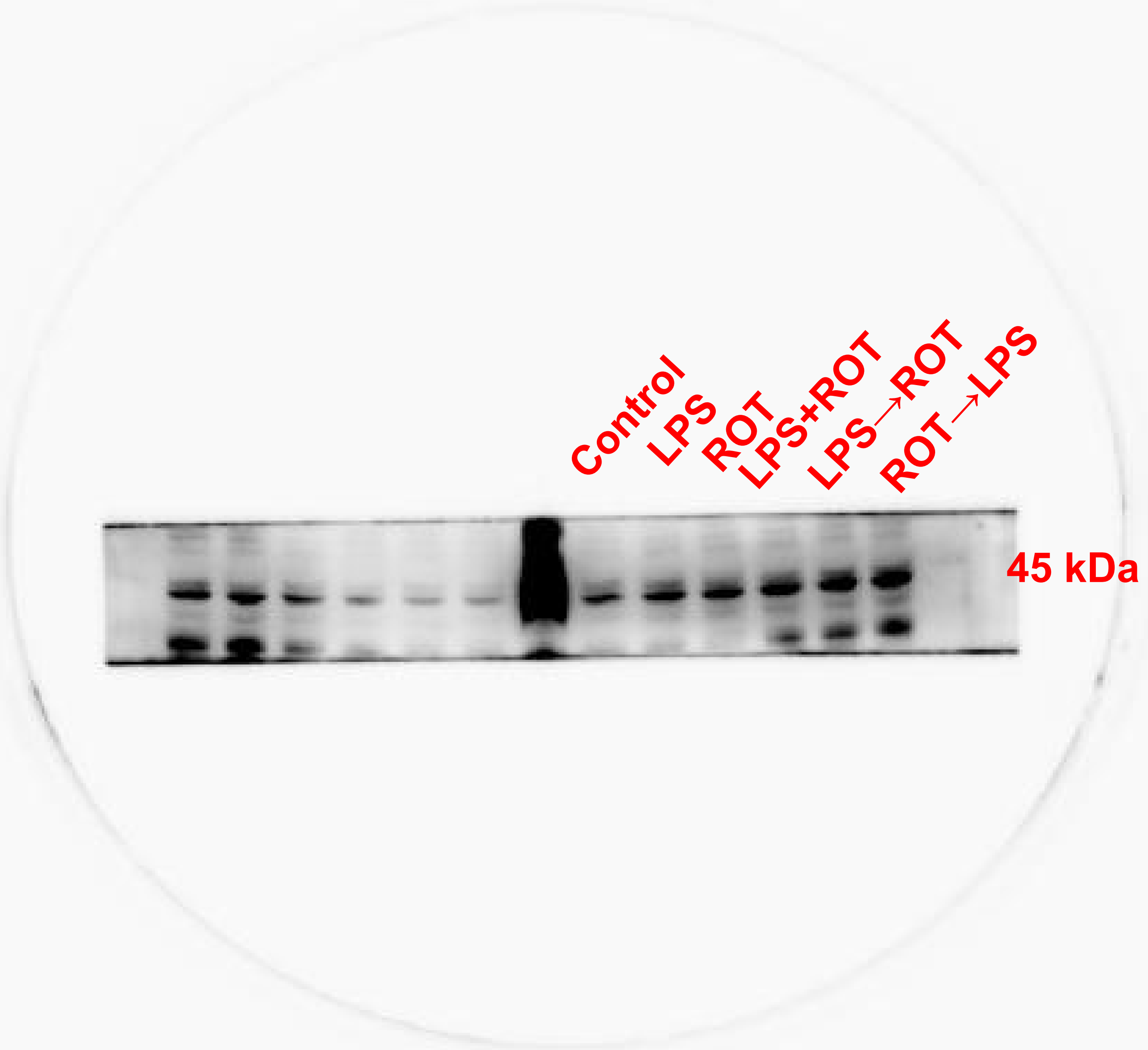

Full unedited gel/blot for
Figure - 4C Arg1

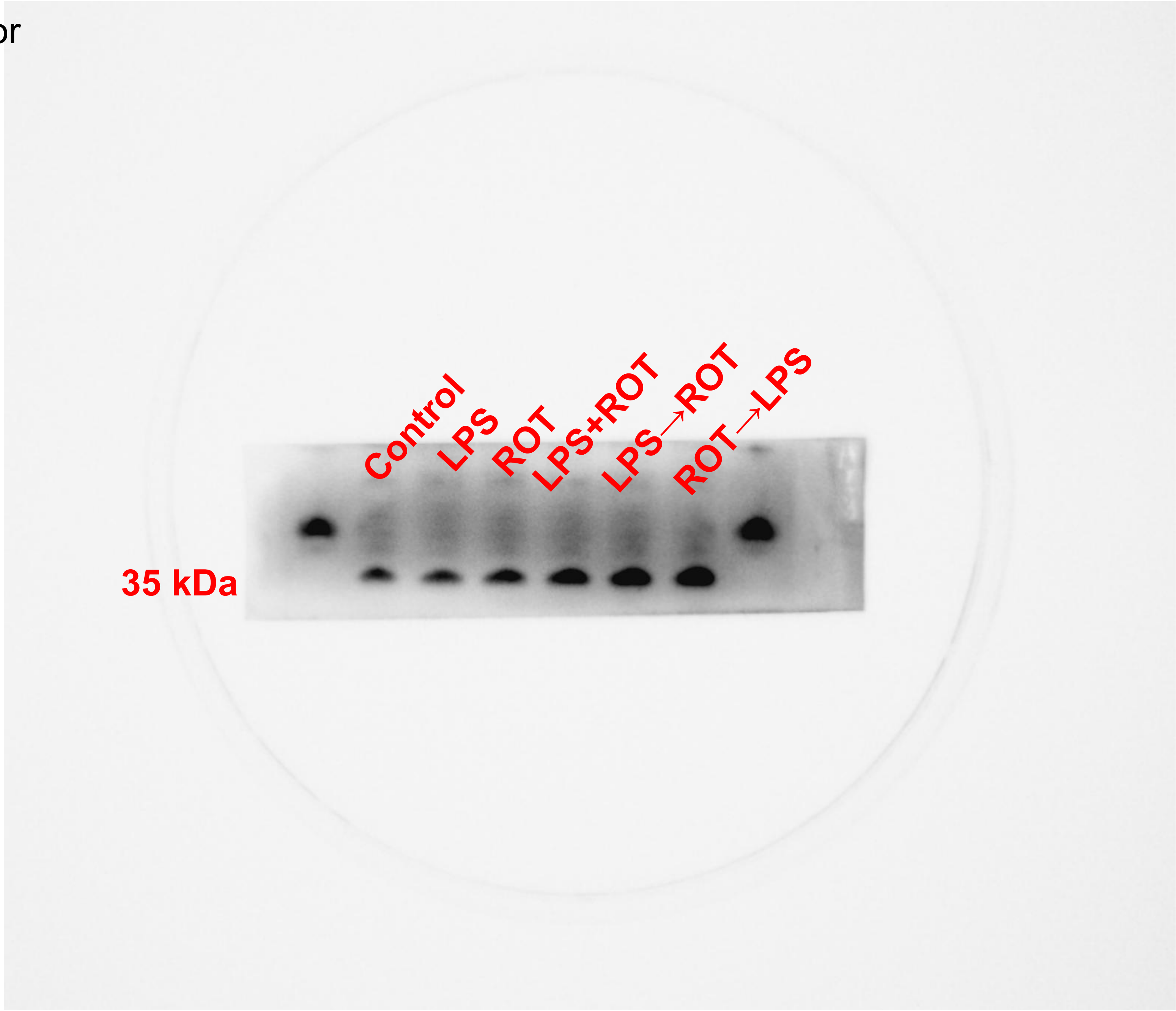

Full unedited gel/blot for
Figure - 5B GFAP

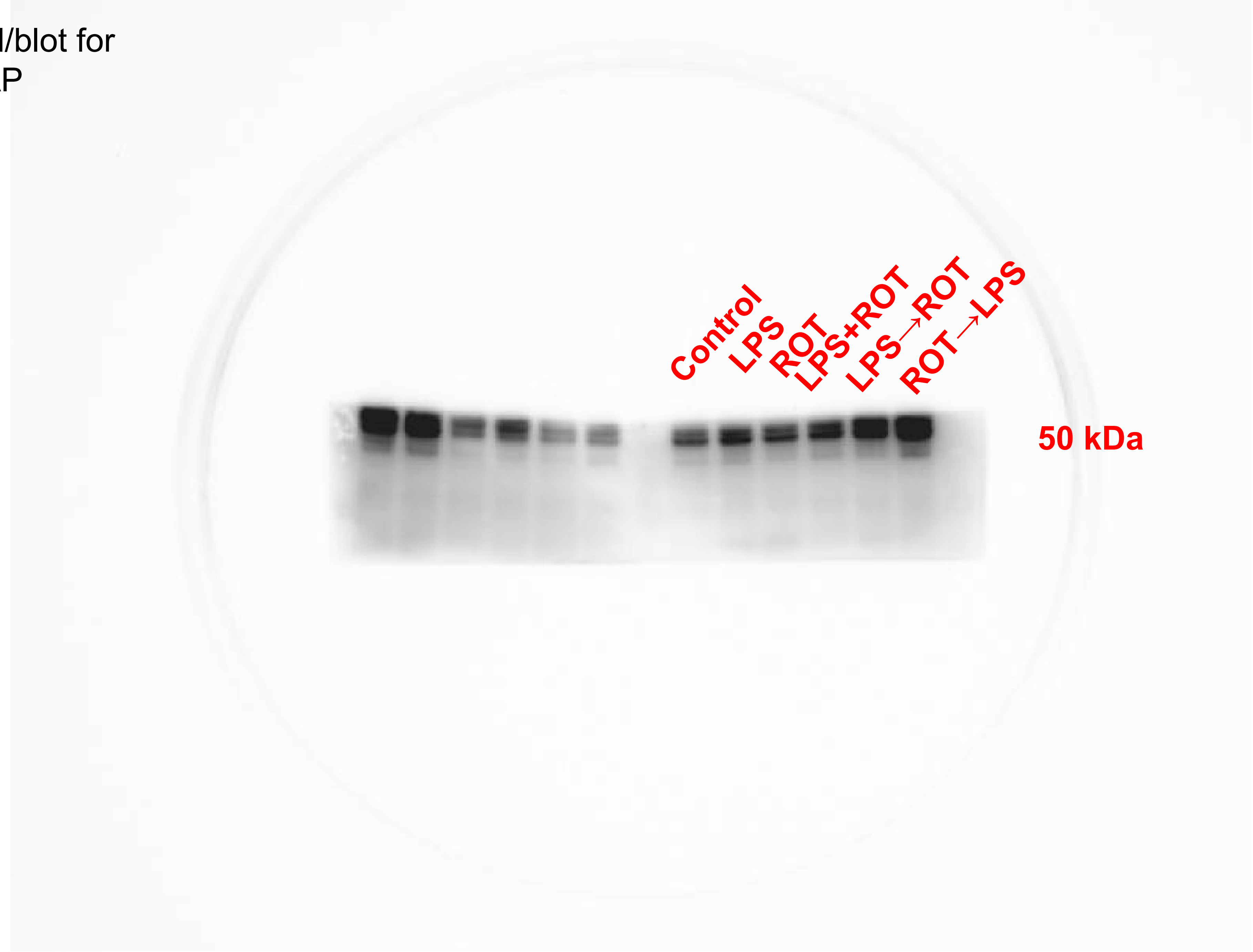

Full unedited gel/blot for
Figure - 5C LCN2

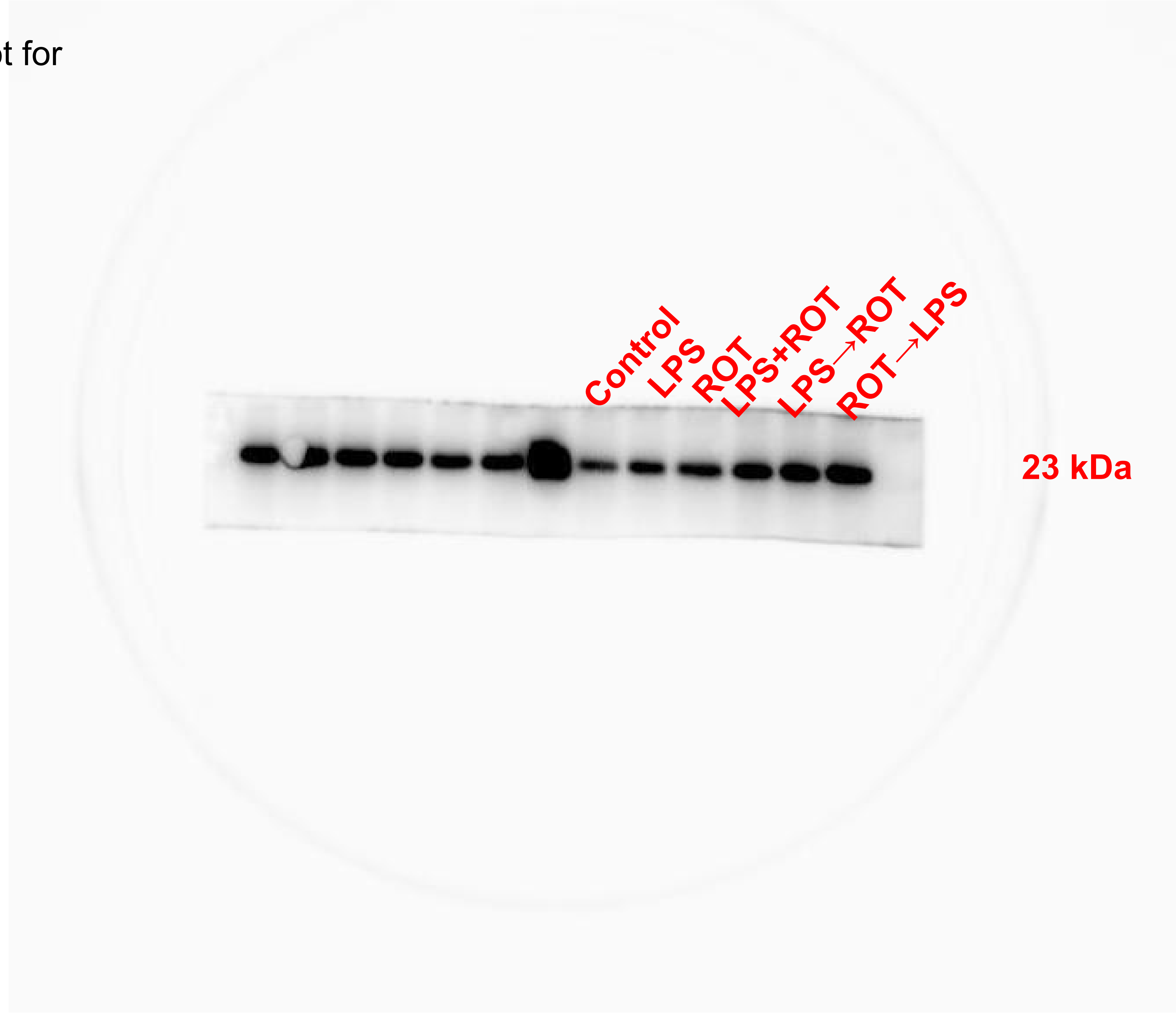

Full unedited gel/blot for
Figure - 5C C3d

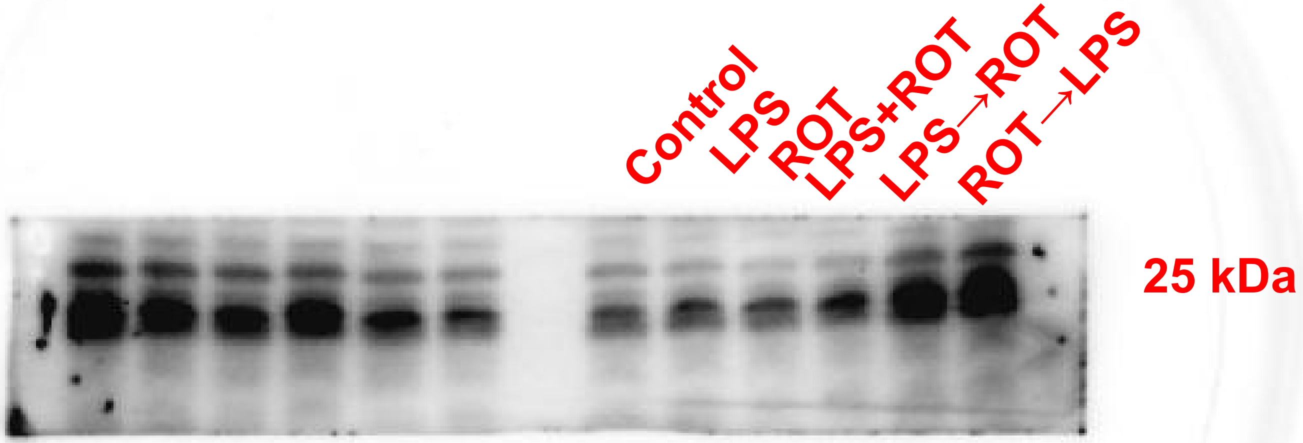

Full unedited gel/blot for
Figure - 5C BDNF

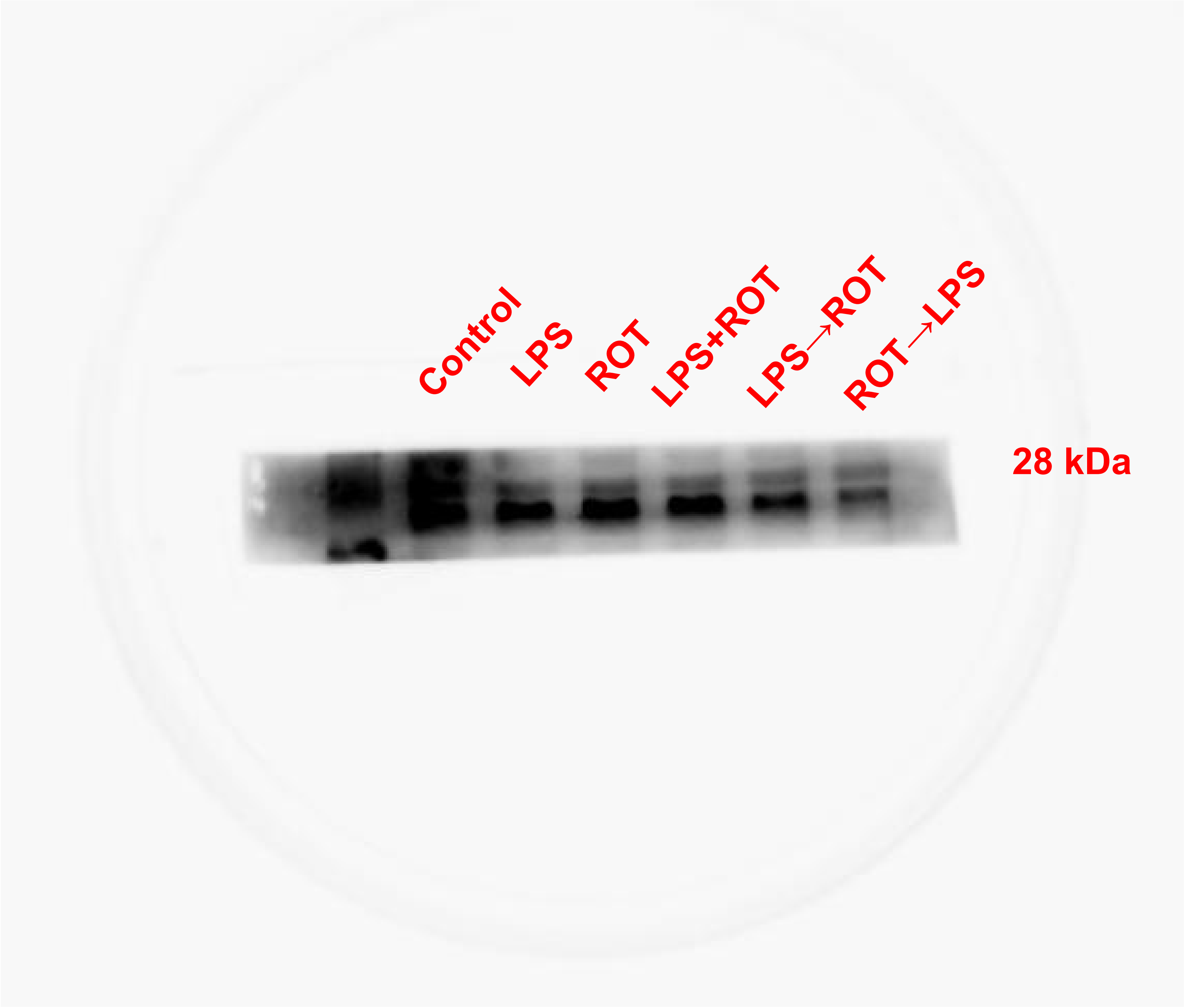

Full unedited gel/blot for
Figure - 5C GDNF

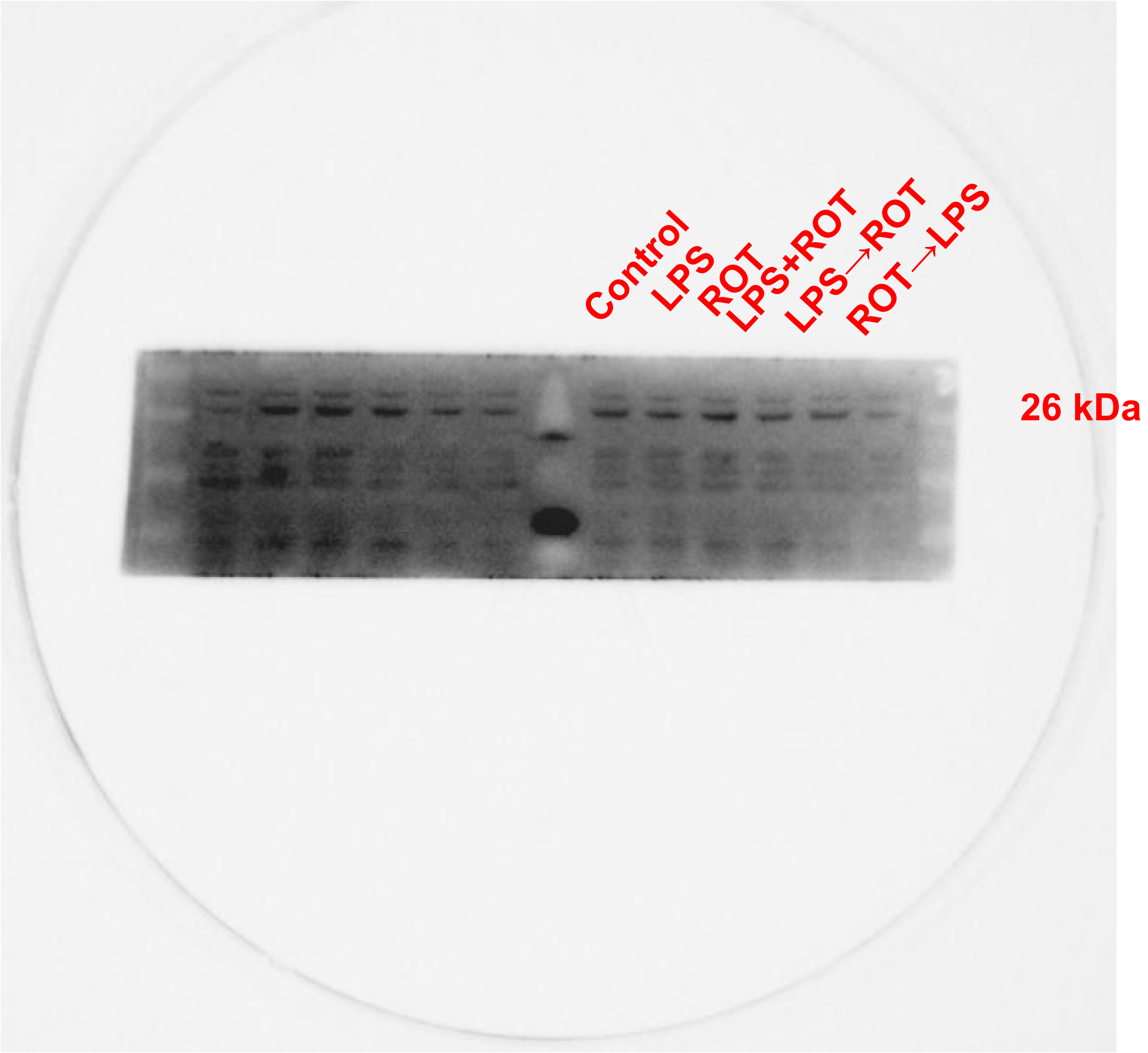

Full unedited gel/blot for
Figure - 6C N-Nrf2

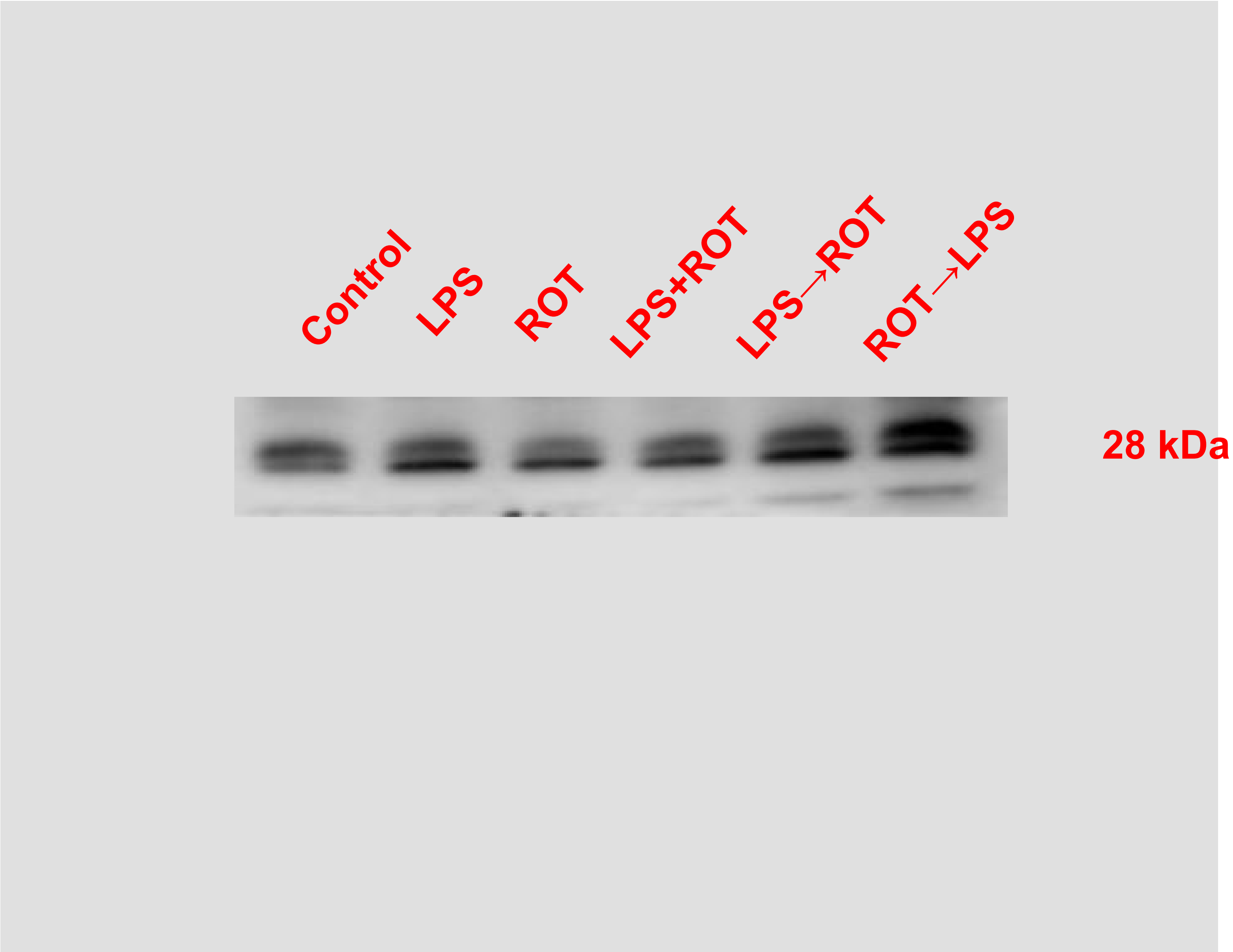

Full unedited gel/blot for
Figure - 6C C-Nrf2

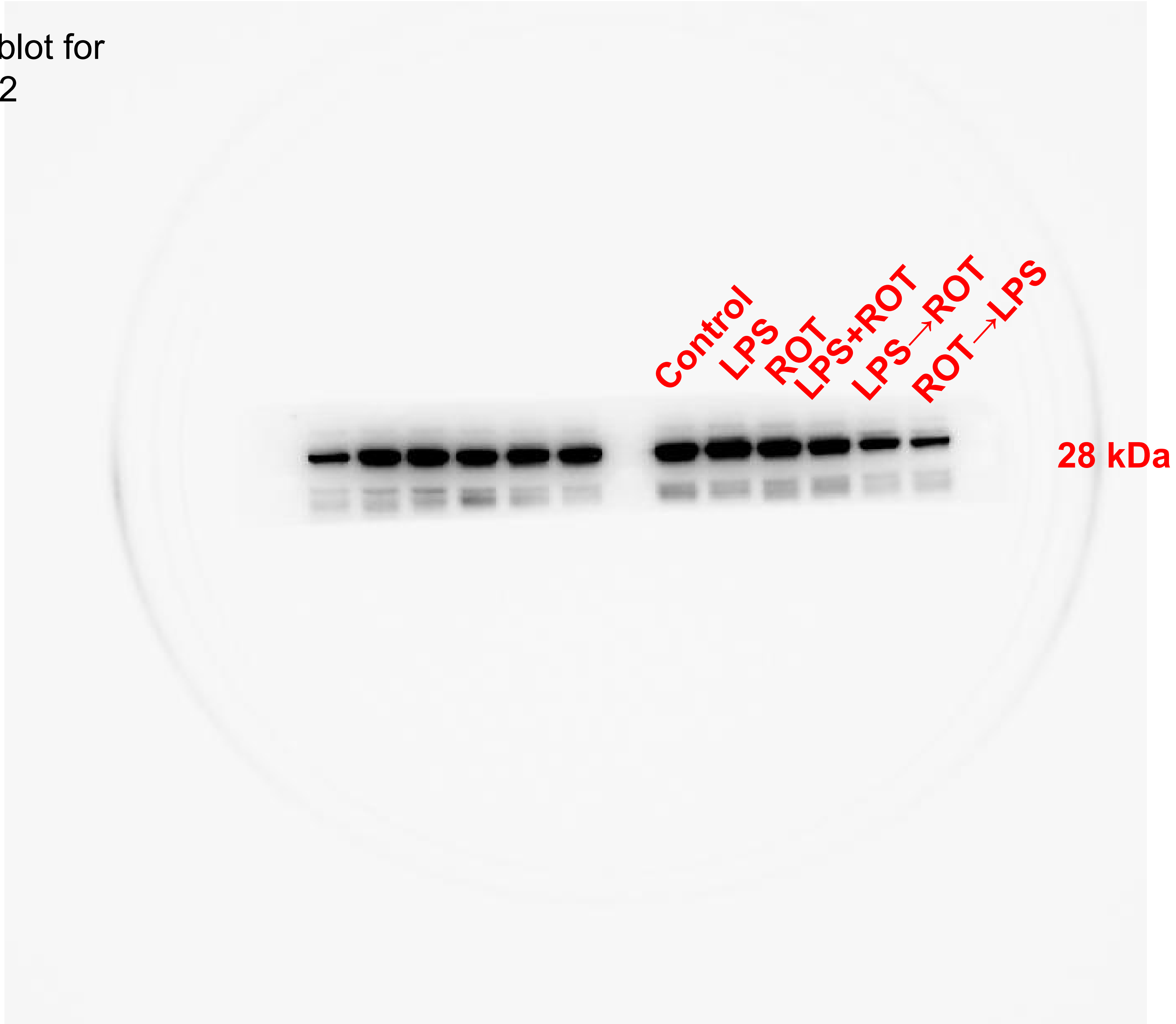

Full unedited gel/blot for
Figure - 6D HO1

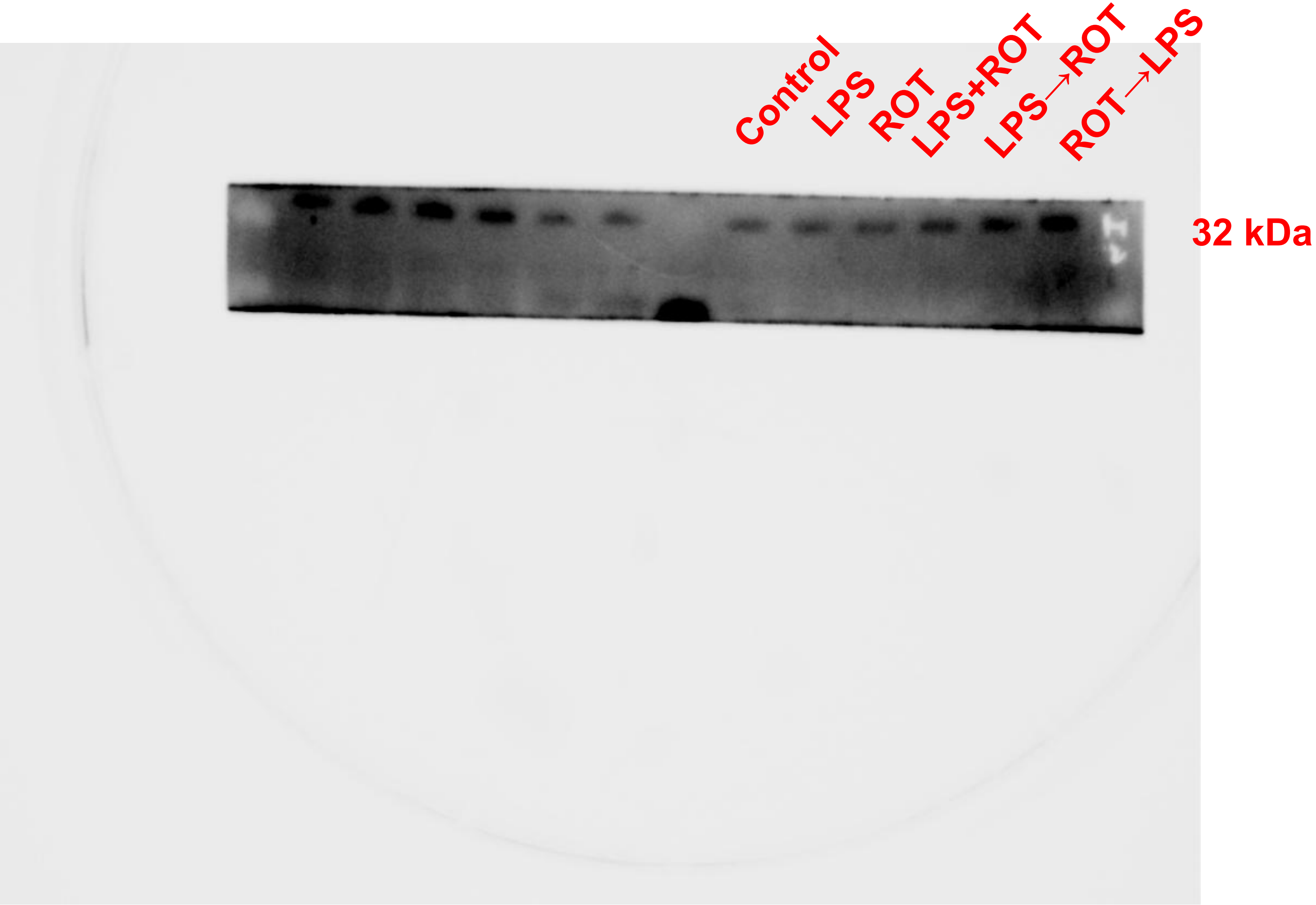

Full unedited gel/blot for
Figure - 6D NQO1

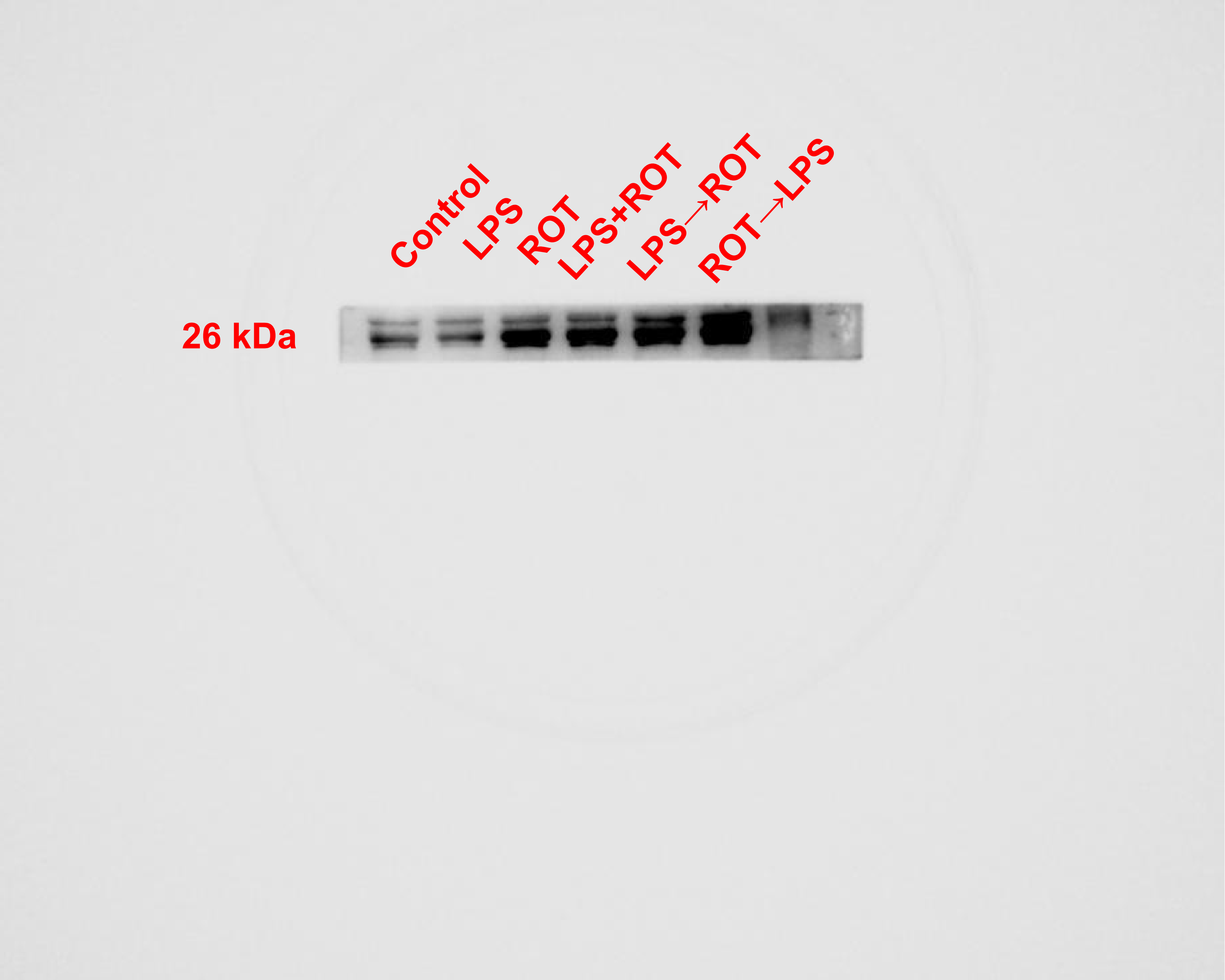

Full unedited gel/blot for
Figure - 6D Keap1

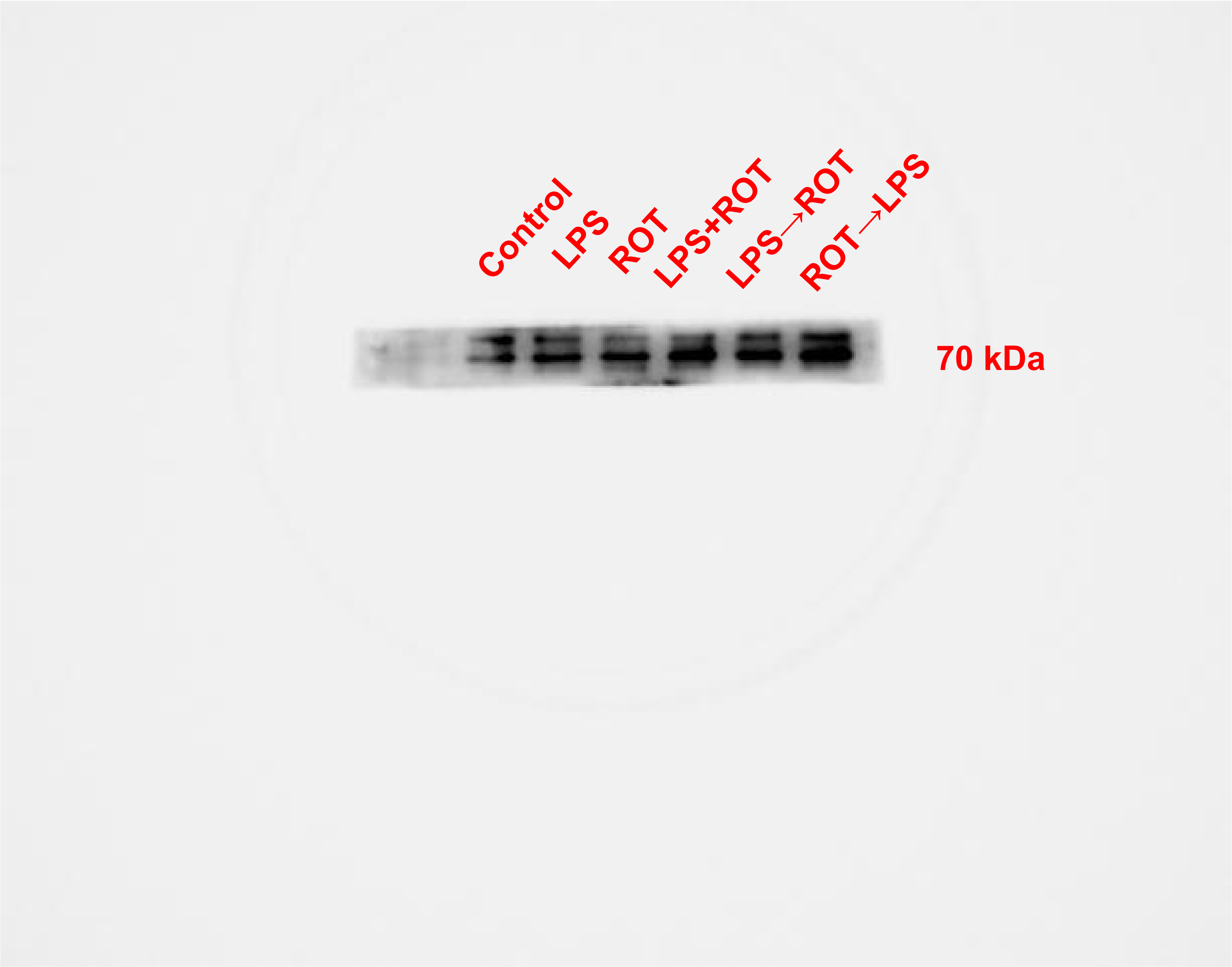

Supplement: Supplementary file 1 — Figure S1 [file CNS-29-2281-s001.pdf]
